# Immunosuppressive tumor microenvironment and immunotherapy resistance of esophageal carcinoma

**DOI:** 10.3389/fimmu.2026.1926551

**Published:** 2026-09-14

**Authors:** Zhenglin He, Hanming Hao, Xinyang Shu, Yifei Ren, Yishuo Ji, Kai Zhao, Yimeng Chen, Liang Han, Yue Hu

**Affiliations:** 1 Department of Biobank, China-Japan Union Hospital of Jilin University, Changchun, China; 2 Department of Biomedical Science, College of Basic Medical Sciences, Jilin University, Changchun, China; 3 School and Hospital of Stomatology, Jilin University, Changchun, China; 4 The First Hospital of Jilin University, Jilin University, Changchun, China; 5 Department of Immunobiology, Yale University School of Medicine, New Haven, CT, United States; 6 Center of Molecular and Cellular Oncology, Yale Cancer Center, Yale University, New Haven, CT, United States; 7 Department of Endoscopy Center, China-Japan Union Hospital of Jilin University, Changchun, China; 8 Department of Pathology, China-Japan Union Hospital of Jilin University, Changchun, Jilin, China

**Keywords:** esophageal carcinoma, tumor microenvironment, immune checkpoint inhibitors, immunotherapy resistance, combination therapy, adoptive cell therapy

## Abstract

Esophageal carcinoma (EC) remains one of the most lethal malignancies, with a five-year survival rate of only 15-25% in advanced stages. Although immunotherapy including immune checkpoint inhibitors (ICIs) and antibody-based therapies have improved clinical outcomes, durable responses are confined to a minority of patients because primary and acquired resistance affect the majority. The tumor microenvironment (TME)—composed of immune cells, stromal cells, and the extracellular matrix—has emerged as a central driver of the immune resistance, wherein infiltrating immune cells and stromal cells form suppressive networks that impair cytotoxic immunity and foster tumor progression. However, a coherent framework linking clinical practice, TME-mediated resistance mechanisms, and therapeutic strategies remains lacking. In this review, we dissect the cellular and molecular basis of TME-driven immune resistance in EC, summarize established and emerging immunotherapies, and evaluate strategies to overcome treatment failure, including TME-targeted therapy, metabolic modulation, photodynamic therapy, and mechanism-guided combination approaches. These insights reframe immunotherapy resistance as a TME-driven process rather than a purely tumor-intrinsic defect, underscoring the need to concurrently target malignant cells and their surrounding immunosuppressive niche. Ultimately, elucidating TME heterogeneity and its dynamic evolution is essential to convert resistant EC into an immunotherapy-responsive disease and to guide precision combination strategies.

## 1 Introduction

Esophageal carcinoma (EC) is an aggressive malignancy with poor long-term survival, particularly in locally advanced, recurrent, or metastatic disease (1, 2). According to the World Cancer Report 2024, EC accounts for 2.4% of all new cancer cases and 4.5% of cancer-related deaths, with five-year survival rates of only 15-25% at intermediate and advanced stages (1, 3). Surgery, radiotherapy, and chemotherapy remain standard treatments, yet durable disease control is rarely achieved (4). Although immunotherapies, particularly immune checkpoint inhibitors (ICIs), have demonstrated significant clinical efficacy across various solid tumors (5, 6), only a subset of EC patients achieves durable benefit (6–8). Primary resistance affects over 60% of patients, while others develop acquired resistance after initial response (9, 10); immune-related toxicities and intertumoral heterogeneity further complicate treatment outcomes (11). Consequently, ICI efficacy in EC is constrained by both incomplete initial sensitivity and adaptive escape during therapy, underscoring the urgent need to elucidate immune resistance mechanisms.

The tumor microenvironment (TME) is increasingly recognized as an interconnected ecosystem driving immunotherapy failure in EC (9, 12). Comprising myeloid subsets, lymphoid populations, stromal components, and extracellular matrix (ECM), the TME forms a self-reinforcing network governed by cytokines, chemokines, immune checkpoints, metabolites, and mechanical signals (13, 14). Furthermore, metabolic reprogramming serves as a shared mechanism through which tumor, stromal, and myeloid cells converge on lymphocyte dysfunction (15). Cancer cells actively reprogram stromal cells to support growth and metastasis (12, 16, 17), while bidirectional cell-stroma interactions promote ECM stiffening and aberrant mechanotransduction, fostering malignant progression (12, 18). Importantly, immunotherapy resistance in EC is now viewed less as an intrinsic tumor cell defect and more as a consequence of TME-mediated immunosuppression, which attenuates tumor immunogenicity and selects for immune-evasive clones (19, 20). Thus, resistance in EC represents an emergent property of a dynamic, crosstalk-driven network.

Recent reviews have profiled selected TME components and the emerging role of immunotherapy in EC (2, 21, 22), yet a unified framework that integrates current clinical evidence, TME-driven resistance mechanisms, and therapeutic strategies remains absent. In this review, we begin by summarizing the current clinical status of EC immunotherapy, and dissect the cellular and molecular basis of TME-mediated immune resistance. Then, we critically discuss therapeutic strategies to overcome these barriers. Finally, we assess emerging immunotherapies and propose potential future directions. By bridging clinical context, biological mechanisms, and emerging therapeutics, this review presents a comprehensive framework for understanding and targeting immune resistance in EC (**Figure 1**).

**Figure 1:**
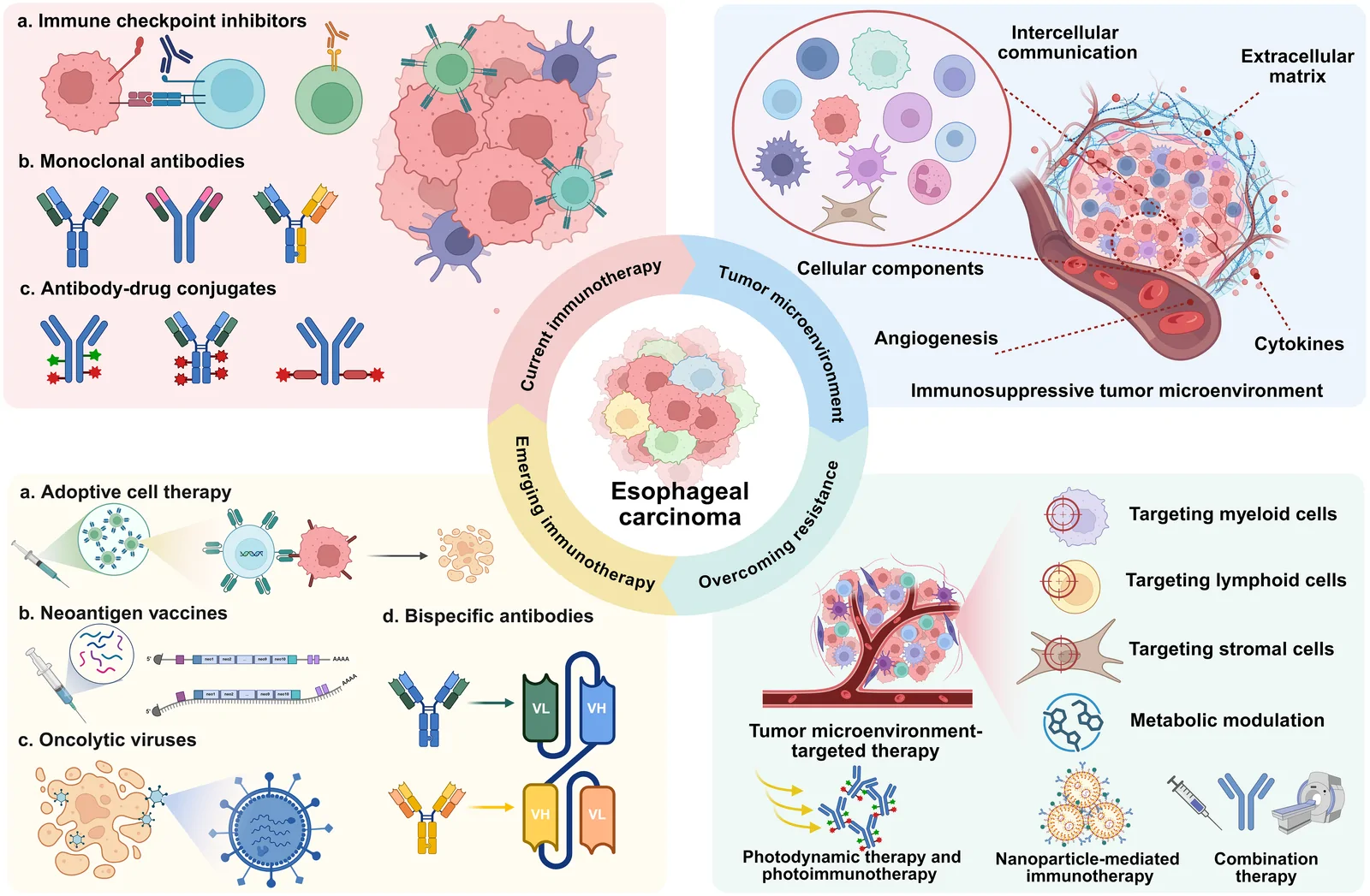
Schematic representations of immunosuppressive TME and immunotherapy resistance of EC. Current immunotherapies include immune checkpoint inhibitors, monoclonal antibodies, and antibody-drug conjugates. TME features include cellular components, angiogenesis, and the extracellular matrix. Strategies to overcome immunotherapy resistance include TME-targeted therapy, photodynamic therapy, photoimmunotherapy, nanoparticle-mediated immunotherapy, and combination therapy. Emerging strategies encompass adoptive cell therapy, neoantigen vaccines, oncolytic viruses, and bispecific antibodies. EC, esophageal carcinoma; TME, tumor microenvironment.

## 2 Clinically established immunotherapies for EC

Tumor immunotherapy harnesses active or passive mechanisms to activate the immune system, enhancing its recognition and elimination of malignant cells (23, 24). This approach has achieved remarkable efficacy in melanoma and non-small cell lung cancer (25), and has driven the clinical development of ICIs and monoclonal antibodies (mAbs) that relieve immunosuppression and potentiate tumor-infiltrating lymphocyte (TIL) cytotoxicity in EC (26, 27). Antibody-drug conjugates (ADCs) further integrate cytotoxic drug delivery with immune activation (28). Collectively, these advances have established immunotherapy as a pivotal therapeutic modality for EC (29–31) (**Table 1**).

**Table 1:** Clinically established immunotherapies for esophageal carcinoma.

Year	Agents/interventions	Cancer subtype	ClinicalTrials.gov ID	Phase	Reference
2019	Nivolumab monotherapy	Advanced esophageal squamous cell carcinoma refractory or intolerant to previous chemotherapy	NCT02569242	III	(32)
2019	Pembrolizumab monotherapy	Advanced, metastatic esophageal adenocarcinoma or squamous cell carcinoma	NCT02559687	II	(33)
2020			NCT02564263	III	(34)
2020	Pembrolizumab and trastuzumab in combination with oxaliplatin or cisplatin, capecitabine or 5-fluorouracil	HER2^+^ metastatic esophageal, gastric, or gastrooesophageal junction cancer	NCT02954536	II	(35)
2021	Pembrolizumab plus cisplatin and 5-fluorouracil	Locally advanced, unresectable or metastatic esophageal squamous cell carcinoma	NCT03189719	III	(6)
2021	Adjuvant nivolumab	Resected esophageal or gastroesophageal junction cancer	NCT02743494	III	(36)
2022	Nivolumab and ipilimumab or nivolumab combined with fluorouracil plus cisplatin	Previously untreated, unresectable advanced, recurrent, or metastatic esophageal squamous cell carcinoma	NCT03143153	III	(37)
2022	Tislelizumab monotherapy	Advanced or metastatic esophageal squamous cell carcinoma	NCT03430843	III	(38)
2023	PD-L1 antibody SHR-1316 combined with carboplatin and etoposide	Locally advanced resectable esophageal squamous cell carcinoma	NCT04215471	II	(39)
2023	Toripalimab combined with definitive CRT	Locally advanced esophageal squamous cell carcinoma	NCT04005170	II	(40)
2024	Capecitabine plus with oxaliplatin over cisplatin plus 5-fluorouracil		NCT02025036	III	(41)
2024	Nivolumab plus ipilimumab or nivolumab in combination with oxaliplatin plus fluoropyrimidine	Previously untreated advanced or metastatic gastroesophageal adenocarcinoma	NCT02872116	III	(42)
2024	Sugemalimab plus cisplatin and plus 5-fluorouracil	Unresectable, locally advanced, recurrent or metastatic esophageal squamous cell carcinoma	NCT04187352	III	(43)
2025	Neoadjuvant serplulimab combined with carboplatin and albumin paclitaxel	Locally advanced resectable esophageal squamous cell carcinoma	NCT05659251	II	(44)
2025	Pembrolizumab combined with paclitaxel/carboplatin or nab-paclitaxel/carboplatin		NCT03792347	I	(45)
			NCT04435197	II	
2025	Camrelizumab plus nab-paclitaxel and cisplatin		NCT05007145	II	(46)
2025	Domvanalimab and zimberelimab plus oxaliplatin, leucovorin, and fluorouracil	Previously untreated advanced HER2^-^ esophageal adenocarcinoma	NCT05329766	II	(47)
2025	Nivolumab combined with cisplatin and paclitaxel	Locally advanced esophageal squamous cell carcinoma	NCT05213312	II	(48)
2025	Zanidatamab plus capecitabine and oxaliplatin	Untreated, metastatic, or advanced HER2^+^ gastrooesophageal adenocarcinoma	NCT03929666	II	(49)
2025	Antibody-drug conjugate IBI343	Advanced gastric or gastroesophageal junction adenocarcinoma	NCT05458219	I	(50)
2025	Antibody-drug conjugate SHR-A1904	Advanced CLDN18.2^+^ gastric or gastroesophageal junction cancer	NCT04877717	I	(51)
2026	Tiragolumab plus atezolizumab, combined with cisplatin and fluorouracil	Previously untreated, locally advanced unresectable or metastatic esophageal carcinoma	NCT03281369	I/II	(52)
	Tiragolumab plus atezolizumab, combined with paclitaxel and cisplatin		NCT04540211	III	(53)
2026	Tislelizumab plus induction and concurrent CRT	Unresectable, locally advanced esophageal squamous cell carcinoma	NCT05520619	II	(54)
2026	Zanidatamab in combination with chemotherapy with or without tislelizumab	HER2^+^ unresectable locally advanced or metastatic gastroesophageal adenocarcinoma	NCT05152147	III	(55)
2026	Nivolumab plus ipilimumab or nivolumab in combination with oxaliplatin plus fluoropyrimidine	Previously untreated, unresectable, HER2^-^ esophageal adenocarcinoma	NCT02872116	III	(56)

### 2.1 ICIs

Under normal physiological conditions, immune checkpoints function to inhibit the proliferation and activation of T cells and other immune cells, thereby maintaining immune homeostasis and preventing autoimmune responses. However, during tumorigenesis, tumor cells can exploit immune checkpoints to induce reduced or even lost activity of immune cells, thereby promoting tumor progression (57). The major immune checkpoint molecules identified to date include PD-1 and its ligand PD-L1, as well as cytotoxic T lymphocyte (CTL)-associated antigen-4 (CTLA-4) (58). The interaction between PD-1 and PD-L1 primarily exerts negative regulation on adaptive immune responses by inhibiting effector T cell (Teff) activity and enhancing the function of immunosuppressive Tregs, thereby maintaining immune homeostasis under normal conditions but promoting tumor immune evasion in malignancy (59). In recent years, PD-1/PD-L1 mAbs have been widely used for the treatment of metastatic EC patients. Pembrolizumab and nivolumab are anti-PD-1 antibodies that block PD-1 binding to PD-L1, disrupting immune checkpoint inhibition, thereby restoring T-cell-mediated tumor killing (60). US Food and Drug Administration (FDA) has approved those ICIs for first-line and second-line EC treatment irrespective of PD-L1 status (61). In esophageal squamous cell carcinoma (ESCC) subgroup, pembrolizumab monotherapy achieved an objective response rate (ORR) of 14.3% in the phase II KEYNOTE-180 trial (NCT02559687), with enhanced efficacy in PD-L1^+^ patients (33). For patients with advanced or metastatic EC, this treatment approach demonstrates durable anti-tumor activity with a manageable safety profile, achieving an ORR superior to that observed in previous third-line treatment regimens. The phase III ATTRACTION-3 trial (NCT02569242) demonstrated that nivolumab significantly improved overall survival (OS) compared with chemotherapy in previously treated advanced ESCC, with fewer grade 3–4 adverse events and better quality of life, establishing it as a standard second-line option (32). Another anti-PD-1 antibody camrelizumab also improves survival rates in pretreated patients with advanced ESCC, supporting further evaluation of the efficacy of PD-1/PD-L1 blockade combined with chemoradiotherapy (31). Anti-PD-L1 antibody atezolizumab binds to PD-L1, blocking its interaction with PD-1 and B7–1 on T cells. This reinstates T-cell activation, proliferation, and anti-tumor immunity, reversing T-cell exhaustion. A phase II proof-of-concept clinical trial demonstrated that atezolizumab achieved encouraging complete response rates in patients with unresectable locally advanced ESCC following radical chemoradiotherapy (EPOC1802) (62). In the following trial (NCT04540211), atezolizumab combined with tiragolumab and chemotherapy significantly improved median progression-free survival (PFS) and OS versus chemotherapy alone, with higher response rates and manageable safety profiles (53). During T cell priming, CTLA-4 expression is markedly upregulated. By binding CD80/CD86, CTLA-4 competitively blocks co-stimulatory signals and generates a negative feedback loop that inhibits T cell activation, thereby promoting tumor growth (63–65). Dual blockade of PD-1/PD-L1 and CTLA-4 exerts synergistic anti-tumor effects across multiple cancer types (66). For example, the phase III CheckMate 648 trial (NCT03143153) demonstrated that nivolumab plus ipilimumab achieved superior overall response rates and durable efficacy in ESCC (37). Despite advances in ICIs for EC (67), over 60% of patients exhibit poor response and limited efficacy, coupled with immune-related adverse events such as myocarditis, pneumonitis, hepatitis, and endocrine toxicities (68, 69).

### 2.2 mAbs

mAbs have fundamentally altered the landscape of oncology by serving as targeted immunotherapeutic agents. These biologics bind with high specificity to discrete antigens expressed on malignant cells, thereby orchestrating an immune-mediated assault on tumor tissue (27). Trastuzumab is a humanized mAb targeting human epidermal growth factor receptor 2 (HER2), which inhibits HER2-mediated cell proliferation and survival signaling, and exerts antibody-dependent cellular cytotoxicity (70). In the ToGA phase III trial (NCT01041404), trastuzumab combined with chemotherapy significantly improved the prognosis of patients with HER2^+^ advanced gastric/gastroesophageal junction adenocarcinoma, as evidenced by prolonged median PFS and OS. Consequently, trastuzumab has been approved for the treatment of HER2^+^ gastroesophageal junction cancer (71). The MAHOGANY trial evaluated an Fc-engineered, anti-HER2 mAb margetuximab plus an anti-PD-1 mAb retifanlimab in HER2^+^ EC. These mAb regimens targeting HER2 and immune checkpoints (PD-1, LAG-3) restored T cell function and enhanced anti-tumor immunity, showing therapeutic potential in advanced disease (72). Sugemalimab is a fully human anti-PD-L1 IgG4 mAb currently undergoing clinical evaluation. It blocks the PD-1/PD-L1 pathway to restore T-cell-mediated anti-tumor immunity and retains FcγRI binding to trigger antibody-dependent cellular phagocytosis of PD-L1^+^ tumor cells by macrophages (73, 74). In the phase III GEMSTONE-304 trial (NCT04187352), first-line sugemalimab plus chemotherapy significantly prolonged PFS and OS in untreated advanced ESCC, with a higher ORR and manageable safety profile (43). Therefore, anti-PD-1/PD-L1 mAbs can effectively treat EC with favorable efficacy and safety profiles. However, the use of mAbs comes with the risk of immune-related responses, including acute anaphylaxis, serum sickness, and the development of anti-drug antibodies (75).

### 2.3 ADCs

ADCs combine mAbs with toxic agents to specifically target antigen-expressing cells. Their efficacy relies on antibody, linker, and payload properties, achieving selective, high-concentration cytotoxic delivery to antigen-overexpressing tumor cells, thereby reducing systemic toxicity (76). Despite approvals for treating drug-resistant cancers, challenges such as limited biomarkers and complex mechanisms remain (77). Trastuzumab deruxtecan (T-DXd) is an antibody-drug conjugate targeting HER2. It delivers a topoisomerase I inhibitor selectively to HER2^+^ tumor cells, inducing potent cytotoxicity and bystander killing in adjacent malignant cells (78). As a second-line treatment, T-DXd regimen demonstrates significantly superior median OS compared to the commonly used ramucirumab plus paclitaxel regimen. T-DXd has been approved by the FDA for patients with HER2^+^, unresectable or metastatic gastric/gastroesophageal junction adenocarcinoma who have previously received trastuzumab therapy (79). Although most ADCs are based on targeting HER2, researchers are still exploring other ADC formulations targeting other targets, such as sacituzumab govitecan and IBI343. In advanced ESCC, combining the FDA-approved TROP2-directed ADC sacituzumab govitecan with the OXPHOS inhibitor IACS-010759 synergistically suppresses tumor growth via PI3K-AKT-mTOR pathway inhibition, supporting clinical evaluation of this strategy in TROP2^+^ ESCC (80). Additionally, Claudin 18.2 (CLDN18.2) aberrancy in gastroesophageal adenocarcinoma renders it a viable target. The ADC IBI343, comprising an anti-CLDN18.2 antibody linked to exatecan, demonstrated manageable toxicity and promising efficacy in a phase I trial (NCT05458219) (50). Furthermore, SHR-A1904, a CLDN18.2-targeted ADC bearing a topoisomerase I inhibitor, demonstrated manageable toxicity and anti-tumor activity in a phase I trial for advanced gastric/gastroesophageal junction cancer (NCT04877717) (51). ADCs combine the advantages of high specificity for targeted delivery with potent cytotoxic activity, making them one of the most prominent therapeutic strategies in anti-cancer drug development (76). Despite their generally favorable tolerability, these ADCs may induce cardiotoxicity, hematologic disturbances, gastrointestinal complications, hepatotoxicity, and oral mucositis, all of which necessitate diligent monitoring and appropriate intervention (81).

## 3 Immunosuppressive TME-mediated immune resistance in EC

### 3.1 Interconnected resistance programs in the EC microenvironment

Immunotherapy resistance in EC is not attributable to a single inhibitory population or pathway, but emerges from an interconnected ecosystem in which tumor cells, myeloid and lymphoid subsets, stromal cells, and the ECM continuously undergo mutual remodeling (**Figure 2**; **Table 2**) (105). This TME-driven resistance can be categorized into interwoven programs: myeloid cell reprogramming, immune checkpoint activation coupled with lymphocyte dysfunction, metabolic dysregulation, stromal and vascular remodeling, and bidirectional cell-cell interactions (106). Collectively, these mechanisms converge on impaired antigen presentation, exclusion of cytotoxic lymphocytes, suppression of T-cell and NK-cell effector functions, and progressive T-cell exhaustion (57). The cellular components described below should be understood as interacting elements within a dynamic resistance network rather than as independent determinants of treatment failure (**Figure 3**).

**Figure 2:**
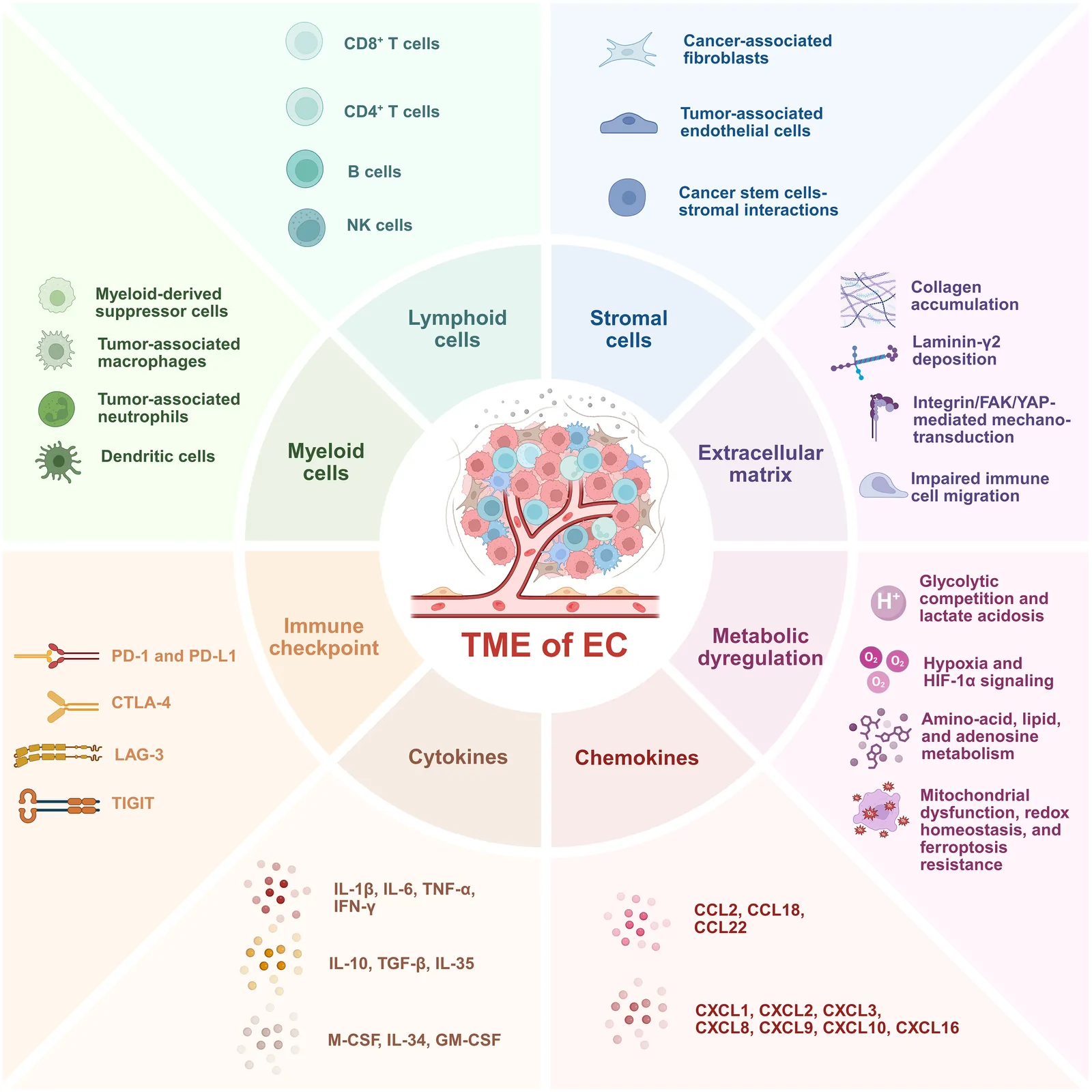
Schematic overview of the TME in EC. The TME of EC comprises dysfunctional lymphoid and myeloid populations, upregulated immune checkpoints, and immunosuppressive cytokine/chemokine networks. Stromal remodeling, ECM deposition, and metabolic dysregulation collectively establish physical and metabolic barriers that drive primary and acquired resistance to immunotherapy. CAF, cancer-associated fibroblast; CCL, C-C motif chemokine ligand; CSC, cancer stem cell; CTLA-4, cytotoxic T-lymphocyte-associated protein 4; CXCL, C-X-C motif chemokine ligand; DC, dendritic cell; EC, esophageal carcinoma; ECM, extracellular matrix; FAK, focal adhesion kinase; GM-CSF, granulocyte-macrophage colony-stimulating factor; HIF-1α, hypoxia-inducible factor-1 alpha; IFN-γ, interferon-gamma; IL, interleukin; LAG-3, lymphocyte-activation gene 3; M-CSF, macrophage colony-stimulating factor; MDSC, myeloid-derived suppressor cell; NK, natural killer; PD-1, programmed cell death protein 1; PD-L1, programmed death ligand 1; TAM, tumor-associated macrophage; TAN, tumor-associated neutrophil; TAEC, tumor-associated endothelial cell; TGF-β, transforming growth factor-beta; TIGIT, T-cell immunoreceptor with Ig and ITIM domains; TME, tumor microenvironment; TNF-α, tumor necrosis factor-alpha; YAP, Yes-associated protein.

**Table 2:** Properties of tumor microenvironment cells in esophageal carcinoma.

Cell type	Markers	Properties in esophageal carcinoma	Reference
MDSCs	EVA1B, ARG1, iNOS	Promote tumor immune evasion, tumor progression, and mediate treatment resistance.	(82)
TAMs	CD163, CD206, CCL2, CCL18, CCL22, MMP9, FOXO1	Promote tumor progression, invasion, and immunosuppression; associated with poor prognosis.	(83–85)
TANs	CD66b	Play key roles in immunosuppression, angiogenesis, tumor invasion and metastasis, and treatment resistance.	(86)
DCs	CLEC9A, CLEC10A, LAMP3	Primarily responsible for antigen presentation and priming naïve T cells.	(87, 88)
NK cells	sMICA/B, CD39, CD49a	Fight against esophageal cancer through direct cytotoxicity, secretion of cytokines, and synergistic adaptive immune enhancement.	(89)
Activated CD8^+^ T cells	CD8, CD39, NKG7, GZMB	Promote tumor immune evasion and disease progression through enrichment in an exhausted state and impairment of cytotoxic functions.​	(90, 91)
Tpex	CD8, CD39, PD-1, TCF1	Play key roles in TLS-mediated immune responses against tumors.	(92)
Tex	CD8, SPRY1	Contribute to proinflammatory phenotype of macrophages and functional state of B cells, which thereby promote anti-tumor immunity by enhancing CD8^+^ T cell effector functions.	(93, 94)
Th	IFN-γ, IL-2, IL-4, IL-5, IL-17	Secrete specific cytokines, regulate other immune cell functions, and interact directly or indirectly with tumor cells.	(95)
Tregs	CD4, CD25, FOXP3, CTLA-4	Exhibit exhaustion and are associated with immune evasion and poor prognosis.	(94, 96)
T_RM_	CD8, CD69, CD103	Associated with a favorable prognosis and response to immunotherapy.	(97, 98)
B cells	CD19, CD20, CD27, CD38	Form TLS and serving as antigen-presenting cells to activate T cell immunity.	(99)
Bregs	CD19, CD24, CD38	Promote immune evasion of esophageal cancer by secreting immunosuppressive cytokines, inducing expansion of other regulatory immune cells, and directly inhibiting effector T cell function.	(100)
CAFs	α-SMA, FAP, PDGFRβ	Promote tumor progression and drive immune exclusion by secreting factors (e.g., TGF-β, CXCL12) and remodeling the extracellular matrix, thereby limiting T cell infiltration.	(101, 102)
TAECs	CD31, PLVAP, VWF	Supply nutrients, highly express angiogenic factors (e.g., VEGF), and regulate immune cell infiltration via adhesion molecules and chemokines.	(103)
CSCs	CD44, ALDH1, EpCAM, SOX2, CD133	Drive tumor initiation, progression, metastasis, treatment resistance and recurrence.	(104)

MDSC, myeloid-derived suppressor cell; TAM, tumor-associated macrophage; TAN, tumor-associated neutrophil; DC, dendritic cell; NK, natural killer; Tpex, progenitor exhausted T cells; Tex, exhausted T cells; Th, T helper cell; Treg, regulatory T cell; T_RM_, tissue-resident memory T cells; Breg, regulatory B cell; CAF, cancer-associated fibroblast; TAEC, tumor-associated endothelial cell; CSC, cancer stem cell; EVA1B, eva-1 homolog B; ARG1, arginase 1; iNOS, inducible nitric oxide synthase; CD, cluster of differentiation; CCL, c-c motif chemokine ligand; MMP, matrix metalloproteinase; FOXO, forkhead box O; CLEC, c-type lectin domain family; LAMP, lysosome-associated membrane protein; sMICA/B, soluble MHC class I polypeptide-related sequence A/B; GZMB, granzyme B; TCF1, T-cell factor 1; SPRY1, sprouty RTK signaling antagonist 1; IFN, interferon; IL, interleukin; FOXP, forkhead box P; CTLA-4, cytotoxic T-lymphocyte-associated protein 4; α-SMA, α-smooth muscle actin; FAP, fibroblast activation protein; PDGFRβ, platelet-derived growth factor receptor β; ALDH1, aldehyde dehydrogenase 1; EpCAM, epithelial cell adhesion molecule; SOX2, SRY-box transcription factor 2; PLVAP, plasmalemma vesicle-associated protein; VWF, von Willebrand Factor; TLS, tertiary lymphoid structures; TGF-β, transforming growth factor-β; VEGF, vascular endothelial growth factor.3.2 Myeloid cell-mediated immunosuppression.

**Figure 3:**
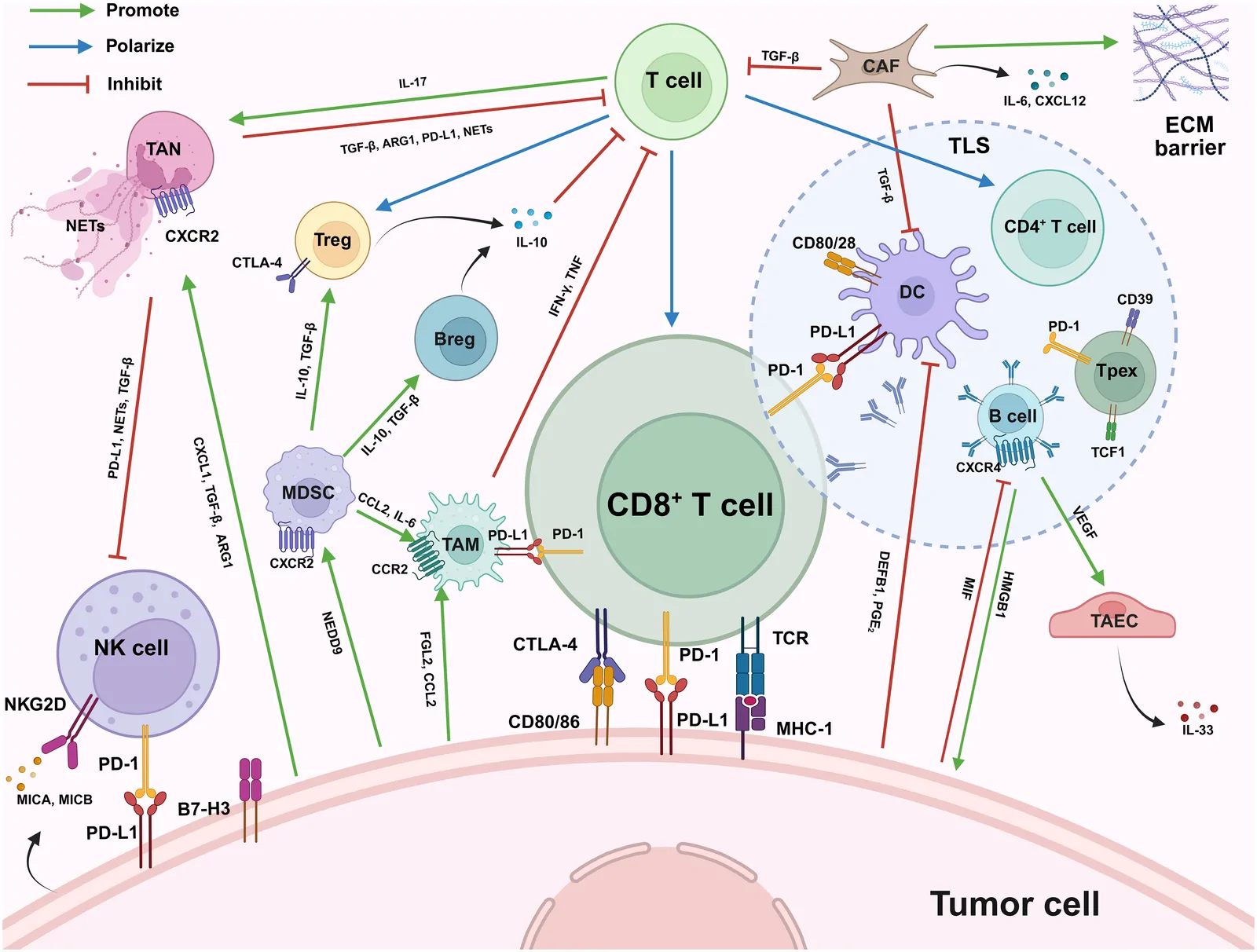
Intercellular crosstalk in the TME of EC. The TME comprises a self-reinforcing network in which myeloid, lymphoid, and stromal cells reciprocally modulate one another via soluble mediators and immune checkpoints. These interactions contribute to the heterogeneous EC microenvironment, driving immune exclusion and therapeutic resistance. ARG1, arginase 1; Breg, regulatory B cell; CAF, cancer-associated fibroblast; CCL2, C-C motif chemokine ligand 2; CCR2, C-C motif chemokine receptor 2; CSF1R, colony-stimulating factor 1 receptor; CTLA-4, cytotoxic T-lymphocyte-associated protein 4; CXCL, C-X-C motif chemokine ligand; CXCR, C-X-C motif chemokine receptor; DC, dendritic cell; EC, esophageal carcinoma; ECM, extracellular matrix; FGL2, fibrinogen-like protein 2; HMGB1, high mobility group box 1; IDO, indoleamine 2, 3-dioxygenase; IFN-γ, interferon-gamma; IL, interleukin; MDSC, myeloid-derived suppressor cell; MHC, major histocompatibility complex; MICA/B, MHC class I polypeptide-related sequence A/B; MIF, macrophage migration inhibitory factor; NET, neutrophil extracellular trap; NK, natural killer; NKG2D, natural killer group 2 member D; PD-1, programmed cell death protein 1; PD-L1, programmed death-ligand 1; PGE_2_, prostaglandin E_2_; PKN2, protein kinase N2; ROS, reactive oxygen species; TAM, tumor-associated macrophage; TAN, tumor-associated neutrophil; TAEC, tumor-associated endothelial cell; TCF1, T-cell factor 1; TCR, T-cell receptor; TGF-β, transforming growth factor-beta; TLS, tertiary lymphoid structure; TNF, tumor necrosis factor; Tpex, progenitor exhausted T cells; Treg, regulatory T cell; VEGF, vascular endothelial growth factor.

### 3.2 Myeloid cell-mediated immunosuppression

#### 3.2.1 MDSC-mediated immune tolerance

MDSCs are a heterogeneous population of immature myeloid cells induced by chronic inflammation (107). Originating in the bone marrow, they migrate to peripheral lymphoid tissues and accumulate in tumor tissues, promoting the formation of the immunosuppressive TME that effectively attenuates the cytotoxic effects against tumor cells (108). The monocyte-derived MDSCs are recruited to the TME and can differentiate into immunosuppressive macrophages. Additionally, MDSCs participate in regulating the function of B cells and T cells by acquiring antigen-presenting phenotypes, thereby suppressing T cell-mediated immune responses and inducing immune tolerance (109).

MDSCs are one of the key immunosuppressive cells in the TME of EC and are associated with poor tumor prognosis (110). MDSCs and the PD-1/PD-L1 axis exhibit close immunosuppressive crosstalk within the TME of EC (13). Yin et al. found that radiotherapy increases PD-L1^+^ MDSCs, while PD-L1 blockade reduces MDSC infiltration, relieves T-cell inhibition, and enhances CD8^+^ T-cell activity. Conversely, fewer MDSCs optimize the microenvironment and boost checkpoint efficacy (111). Elevated circulating MDSCs with high PD-L1 expression suggest MDSCs suppress immunity via the PD-1/PD-L1 pathway (112). Additionally, interleukin-6 (IL-6) also plays a critical role in the recruitment of MDSCs and enables them to exhibit significantly higher levels of reactive oxygen species (ROS) and ARG1, leading to a TME conducive to tumor growth and treatment resistance (113). In ESCC, IL-6 binds human monocytes receptors and directly activates STAT3 signaling. Exosomal miR-21 enters monocytes, inhibits phosphatase and tensin homolog (PTEN), activates nuclear factor kappa B (NF-κB), and triggers autocrine IL-6 to further boost STAT3. These mechanisms drive monocytes to differentiate into monocytic MDSCs with strong immunosuppressive function, which promote cisplatin resistance in ESCC (114). CD38 is highly expressed on MDSCs and functions as both a metabolic regulator and an immune checkpoint, thereby coordinating the immunosuppressive network (115). Karakasheva et al. found that CD38^+^ MDSCs were more immature, showed stronger T-cell suppression, and promoted faster tumor growth than CD38^-^ MDSCs. Mechanistically, tumor-derived factors including IL-6, insulin-like growth factor binding protein 3 (IGFBP-3), and C-X-C motif chemokine ligand 16 (CXCL16) induced CD38 expression. CD38 signaling activated NF-κB, which boosted inducible nitric oxide synthase (iNOS) production essential for immunosuppression (116). Furthermore, ESCC marker NEDD9 is a member of the signaling mediator Crk-associated substrate (Cas) family, which participates in various biological processes, including cell adhesion, motility, cell cycle regulation, apoptosis, and tumorigenesis (117). NEDD9 can regulate the expression of CXCL8 through the extracellular signal-regulated kinase (ERK) pathway, recruiting MDSCs into the tumor, thereby MDSC promotes the stemness of ESCC cells (118). Additionally, MDSCs suppress T and NK cell function in ESCC via ROS, peroxynitrite, TCR/CD8 phosphorylation, and methylglyoxal transfer, driving tumor progression (119). MDSCs cooperate with TAMs and Tregs through IL-10, TGF-β, chemokine and checkpoint signaling, while simultaneously depriving effector lymphocytes of nutrients and generating oxidative stress (13). Therefore, targeting MDSCs represents a promising approach to reverse immunosuppression (120). Future research should further distinguish MDSC subpopulations and their molecular pathways, enabling targeted immunotherapies that reverse immunosuppression and improve patient outcomes in EC.

#### 3.2.2 TAMs and immune microenvironment regulation

TAMs play crucial roles in promoting EC invasion, metastasis, drug resistance, EMT, and immune evasion (83). Conventional studies investigating the immunopathology mechanisms of EC have predominantly employed the macrophage polarization binary model, which defines pro-inflammatory M1 macrophages and anti-inflammatory M2 macrophages as two opposing functional states (121). In this regard, M2-polarized TAMs deplete CD8^+^ T cells via the PD-1/PD-L1 axis, reducing anti-tumor immunity and accelerating EC progression (57, 122). Besides, multiple chemokines, cytokines, and immune checkpoints orchestrate TAM-mediated immunosuppression in EC (106, 123). The C-C motif chemokine ligand 2 (CCL2)-CCR2 axis recruits TAMs to the TME and drives M2 polarization, enhancing ESCC cell proliferation, invasion, and epithelial-mesenchymal transition (EMT) while upregulating PD-L2 to facilitate immune evasion through PD-1 signaling (124, 125). Conversely, LINC00330 suppresses CCL2, reprogramming TAMs toward the M1 phenotype and reversing pro-tumor effects (126). CCL22, another TAM-derived chemokine in ESCC (127), promotes diacylglycerol kinase α (DGKα)-mediated phosphatidic acid generation, thereby attenuating intratumoral ROS production and fostering immune resistance (128). Furthermore, CSF-1 binding to CSF-1R similarly skews TAMs toward an M2-like state (129); blockade of this axis enhances CD8^+^ T cell infiltration and curtails M2 polarization (130). TAM-derived cytokines such as interferon-γ (IFN-γ) and tumor necrosis factor (TNF) upregulate tumor cell PD-L1, which in turn drives adaptive macrophage resistance to IFN-γ, creating a vicious cycle that exacerbates disease and induces primary ICI resistance (131). PD-L1 enforces an immunosuppressive TAM phenotype associated with poor prognosis. In the absence of PD-L1, STAT1/NF-κB p65 phosphorylation and p-STAT6 downregulation shift macrophages toward M1 polarization (59). Therefore, TAMs can leverage the PD-1/PD-L1 axis to construct an immunosuppressive microenvironment, whereas anti-PD-1 therapy not only helps suppress T-cell immune checkpoints but also promotes the repolarization of TAMs, thereby enabling more effective competition with EC cells (132).

Notably, increasing evidence from studies of the tumor immune microenvironment indicates that macrophage states in EC extend well beyond the classical M1/M2 classification. Newly identified TAM subpopulations, such as inflammatory (Inflam-TAMs), proangiogenic (Angio-TAMs), lipid-associated (LA-TAMs), and immunoregulatory (Reg-TAMs), all influence immunotherapy responsiveness of EC (133). Mechanistically, inflam-TAMs express IL-1β, CXCL1/2/3/8, and CCL3, which cooperatively amplify local inflammation and recruit granulocytes, monocytes, and lymphocytes to shape tumor-associated immune responses in EC (134, 135). Moreover, angio-TAMs exhibit high expression of angiogenic signature markers such as VCAN, FCN1, and THBS1. Previous studies have demonstrated that Angio-TAMs are typically enriched in hypoxic regions of the TME across various human cancers including EC (136). LA-TAMs exhibit expression profiles characteristic of lipid-related genes such as APOC1, APOE, ACP5, and FABP5, demonstrating significant enrichment within lipid metabolism pathways (137, 138). Dai et al. revealed that chromosomal instability in ESCC reshapes the tumor immune microenvironment by enriching LA-TAMs, which correlate with CD8^+^ T-cell exhaustion, upregulated immunosuppressive markers (TREM2, LGALS3/1), and reduced immune infiltration, fostering a pro-metastatic niche (138). In Reg-TAMs, the expression levels of ARG1, MRC1, and CX3CR1 are elevated (139–141). Future research should focus on characterizing the continuous diversity of TAMs, as well as the specific functions and regulatory mechanisms underlying their subpopulations in EC.

#### 3.2.3 TANs plasticity and NET-mediated resistance

Neutrophils are the first line of defense against pathogens and the most abundant immune cells in the TME (142). TANs polarize into anti-tumor N1, pro-tumor N2 phenotypes, and polymorphonuclear MDSCs (PMN-MDSCs) (143, 144). In advanced EC, N2 TANs further inhibit T and NK cell functions via PD-L1 expression while recruiting Tregs and M2 macrophages to establish an immunosuppressive milieu (145, 146). Multiple signaling axes recruit and functionally shape TANs to build an immunosuppressive microenvironment in solid tumors including EC (146). CXCR2, a critical G protein-coupled receptor (GPCR) mediating TAN recruitment and activation, is highly expressed in ESCC and correlates with lymph node metastasis and reduced OS. CXCR2 upregulates TGF-β and ARG1 to drive TAN generation and tumor progression (147, 148). Inhibiting CXCR2 may therefore reduce TAN infiltration and enhance immunotherapy efficacy. Additionally, IL-17, a pro-inflammatory cytokine produced by activated T cells, binds IL-17R to promote CD8^+^ T cell inactivation and reduce sensitivity to PD-1/CTLA-4 blockade. During the initial stages of tumor formation, elevated IL-17 enhances neutrophil infiltration, further strengthening immunosuppression and diminishing ICI efficacy (149). In contrast, IL-17^+^CD4^+^ T cells stimulate tumor cells to secrete CXCL2 and CXCL3, thereby recruiting myeloperoxidase^+^ (MPO^+^) neutrophils, potentiating the neutrophil-mediated anti-tumor immunity within ESCC tumor nests by promoting the production of cytotoxic molecules including ROS, MPO, TNF-related apoptosis-inducing ligand (TRAIL), and IFN-γ (143). Blocking the IL-17/IL-17R axis with neutralizing antibodies may restore CD4^+^ and CD8^+^ T cell abundance and activation, thereby improving immunotherapeutic outcomes.

Neutrophil extracellular traps (NETs), released through unique neutrophil death, are bactericidal mesh-like structures that capture pathogens and promote tumor metastasis (150). Within the TME, NETs encapsulate tumor cells, forming physical barriers that block immune cell access and capture circulating tumor cells to promote dissemination (151, 152). NETs also induce CD8^+^ T cell exhaustion, promote TGF-β secretion to suppress NK cell cytotoxicity, and thereby regulate immune evasion (153). B7-H3 (CD276), highly expressed in ESCC and associated with poor prognosis, modulates NET formation via the CXCL1/CXCR2 pathway while suppressing NK activity to promote tumorigenesis (154). NETs not only promote distant tumor metastasis but also induce resistance to conventional therapies by forming physical barriers and fostering an immunosuppressive microenvironment (155). Therefore, targeting NETs may represent a potential therapeutic strategy for EC. However, since neutrophils exhibit anti-tumor and pro-tumor heterogeneity, bulk RNA sequencing cannot fully capture this complexity, and single-cell RNA sequencing faces technical challenges due to low RNA levels and short lifespan. Consequently, more sophisticated approaches are required to elucidate their dual role (146, 156).

#### 3.2.4 Dysfunctional antigen presentation by DCs

As antigen-presenting cells (APCs), DCs can interact with a variety of immune cells and serve as a bridge between the innate and adaptive immune responses (57). DCs are crucial components of the TME, capable of delivering molecular markers and metabolic information from tumor cell surfaces to T cells, thereby triggering T cell activation and immune responses (157). This process generates a substantial number of CTLs, which subsequently eliminate tumor cells (158). The maturation and activation of DCs are crucial for their immune-stimulatory function. Immature DCs exhibit high endocytic activity and upregulated inhibitory receptors, making them incapable of activating T lymphocytes, thereby promoting immune tolerance (159).

DCs play an important role in immunotherapy resistance in EC through crosstalk with other immune cells (160). As key targets of PD-L1-blocking antibodies, DCs express PD-L1 at higher levels than CD80 on both peripheral and tumor-infiltrating subsets in cancer patients. PD-L1 can bind PD-1 and CD80; blockade of PD-L1 on DCs alleviates cis-mediated sequestration of CD80, thereby restoring CD80/CD28 co-stimulatory interactions that enhance T-cell priming (161, 162). Tumor-derived defensin beta 1 (DEFB1) inhibits DC maturation and recruits immature DCs that fail to effectively activate CD8^+^ T cells. Consequently, CD8^+^ T-cell infiltration and cytotoxicity are reduced, forming an immunosuppressive TME in ESCC (163). Moreover, cancer-associated fibroblast (CAF)-secreted TGF-β impairs DC maturation and inhibits Treg differentiation, whereas tumor-secreted prostaglandin E_2_ (PGE_2_) reduces cytokine and receptor expression in both NK cells and DCs to promote immune suppression (164, 165). Zhu et al. reported that EZH2 drives a CD8^+^ T-cell desert phenotype in ESCC by suppressing CXCL9 expression and DC recruitment. Pharmacological targeting of EZH2 in tumor cells reshapes NF-Κb-mediated CXCL9 transcriptional activation and promotes DC maturation by reducing vascular endothelial growth factor C (VEGFC) secretion, thereby enhancing cytotoxic CD8+ T-cell infiltration into the TME and curtailing tumor immune evasion (166). Thus, DCs within the TME of EC influence clinical prognosis by modulating immune cell activity. However, the expression patterns and functional roles of distinct DC subsets in EC tissues remain to be fully characterized.

Collectively, these myeloid networks establish a self-reinforcing immunosuppressive ecosystem. Dissecting their heterogeneity and crosstalk with checkpoints is essential for designing rational combination strategies to overcome resistance and improve EC immunotherapy outcomes.

### 3.3 Immune checkpoint activation and lymphocyte-mediated immune resistance

Lymphocyte-mediated immune resistance in EC arises from multifaceted dysregulation across adaptive and innate compartments. Impaired CD8^+^ T-cell recruitment, Treg/Breg immunosuppression, and NK-cell dysfunction collectively cripple anti-tumor immunity. Mechanisms such as checkpoint activation, cytokine expression, and immune crosstalk can drive exhaustion or phenotypic plasticity, thereby enabling tumor persistence and immunotherapy failure. Targeting these lymphocyte-intrinsic and -extrinsic barriers is crucial for restoring effective immune surveillance.

#### 3.3.1 Impaired CD8^+^ T-cell recruitment and activation

CD8^+^ T cells are the principal end effectors of anti-tumor immunity, and their intratumoral infiltration correlates with prognosis across multiple malignancies including EC (167). Naïve T cells undergo activation and differentiate into CD8^+^ T cells upon receiving TCR signaling, co-stimulatory cues, and cytokine signals triggered by antigen-MHC engagement (168, 169). Activated CD8^+^ T cells recognize and eliminate tumor cells bearing cognate antigens (169). In ESCC, the abundance of tumor-infiltrating CD8^+^ CTLs is governed by multiple regulatory layers (123). The oncogenic lncRNA FOXP4-AS1 is highly expressed in ESCC and enriches H3K4me3 and MLL2 at the FOXP4 promoter, thereby suppressing CTL survival and accelerating PD-L1-mediated exhaustion and immune evasion (170). Robust cytotoxic CD8^+^ T cell infiltration generally predicts favorable therapeutic responses, whereas an immune-desert TME lacking CD8^+^ T cells portends poor clinical outcomes (171, 172). Mechanistically, elevated EZH2 in immune-desert tumors catalyzes repressive histone trimethylation at the NF-κB-mediated CXCL9 promoter, silencing CXCL9 transcription and preventing CTL recruitment. EZH2 targeting can restore this axis, reduce VEGF secretion to promote DC maturation, and enhance CTL infiltration (166). Furthermore, alleviating checkpoint-mediated interactions between stressed epithelial tumor cells and CD8^+^ T cells prevents T cell depletion and restores anti-tumor activity (173). Therefore, tumor cells impair the recruitment and activation of CD8^+^ T cells within the TME of EC. Restoring chemokines or relieving checkpoint signals can reinvigorate CTL infiltration and cytotoxicity, offering rational strategies to overcome immune evasion and improve immunotherapy outcomes.

#### 3.3.2 Immune suppression versus T-cell exhaustion

Immunosuppression can be induced by cells (e.g., Tregs, MDSCs, and TAMs) and factors (e.g., TGF-β, IL-10, and adenosine), exhibiting a certain degree of reversibility (174). In contrast, T-cell exhaustion represents a differentiated state resulting from sustained antigenic stimulation, involving signaling pathways such as T-cell factor 1 (TCF1), thymocyte selection-associated high mobility group box (TOX), orphan nuclear receptor 4A (NR4A), and CD39 (175–178). Importantly, non-terminally exhausted CD8^+^ T cells—unlike their terminally exhausted counterparts—are associated with superior prognosis and heightened ICI responsiveness, suggesting that preserving or restoring an early exhaustion state may represent a viable immunotherapeutic strategy (168).

Progenitor exhausted T cells (Tpex), defined by co-expression of TCF-1 and PD-1, retain stem-like self-renewal and proliferative capacity (179). Tpex, enriched in both tumor-draining lymph nodes and intratumoral niches, serve as a renewable reservoir that undergoes clonal expansion upon ICI treatment and differentiates into effector-like cells to sustain durable anti-tumor immunity (180). Clinically, higher intratumoral or nodal Tpex abundance consistently predicts superior ICI response and improved prognosis across malignancies (180, 181). In ESCC, single-cell profiling identified a progenitor-like exhausted subset marked by SPRY1 (CD8^+^ Tex-SPRY1) that is associated with complete remission following immune checkpoint blockade (ICB) (93). Mechanistically, SPRY1 enforces the progenitor phenotype and enhances ICB efficacy by remodeling the microenvironment toward proinflammatory macrophages and functional B cells, thereby augmenting CD8^+^ effector functions (93). Concordantly, imaging mass cytometry revealed that CD39^+^PD-1^+^TCF1^+^ Tpex cells are enriched in tertiary lymphoid structures (TLS) and positively correlate with ICB benefit; circulating Tpex further expand in responders post-therapy (92). In contrast, terminally exhausted T cells (Tex) downregulate TCF-1 and display high TOX, CD39, and co-inhibitory receptor expression (92, 176). Tex exhibit proliferative arrest, diminished cytokine production, and epigenetic fixation of the exhausted state, rendering them largely refractory to ICI therapy (182). Transcriptomic analyses in EC have further stratified tumors by Tex-related signatures, demonstrating that elevated Tex burden correlates with poor survival, suppressed immune infiltration, and immunotherapy resistance (183). Therefore, therapeutic strategies preserving the Tpex pool, promoting Tpex expansion, or blocking terminal exhaustion represent promising avenues to enhance immunotherapy efficacy in EC.

#### 3.3.3 Tregs/Bregs and immunosupression

Tregs with immune-suppressive characteristics can impair the effects of APCs by highly expressing CTLA-4, which competitively binds to CD80/CD86 on APCs (184, 185). They suppress Teffs, DCs, and macrophages via inhibitory checkpoints (CTLA-4, GITR, PD-1) and cytokine secretion (IL-10, TGF-β), thereby promoting immune evasion and immunotherapy resistance in EC (186). Notably, ESCC tumors harbor significantly higher Treg infiltration than normal esophageal mucosal tissues. Tregs express ectonucleotidases CD39 and CD73, which sequentially hydrolyze extracellular ATP to adenosine, which engages the A2A receptor on CD8^+^ T cells to inhibit cytokine production and suppress proliferation and effector functions of T, B, and NK cells (187, 188). A2A activation further dampens MAPK signaling while upregulating TGF-β, FOXP3, and immune checkpoints (PD-1, CTLA-4, LAG-3), thereby facilitating tumor escape (188). Concurrently, sustained NF-κB activation induces pro-inflammatory chemokines (e.g., CCL2) and PD-L1 expression, recruiting Tregs and MDSCs to establish a TME unfavorable for Teff function (189). Never in mitosis A-related kinase 7 (NEK7), a serine/threonine kinase critical for tumor and microenvironment crosstalk (190), positively correlates with immune cell infiltration in EC models. Wang et al. demonstrated that NEK7 knockout reduces Treg infiltration, blocks tumor growth, and prolongs survival via the NLRP3/PD-L1 axis, establishing NEK7 as a critical node connecting innate sensing to Treg-mediated checkpoint resistance (190).

Within the TME, B cells exert anti-tumor effects by recognizing diverse tumor antigens through immunoglobulins (Igs) (191, 192). Conversely, Bregs suppress immune responses through cytokine secretion and paracrine interactions, attenuating effector responses and promoting tumor progression (192–194). Upon infiltration into the TME, Bregs establish an immunosuppressive niche by secreting IL-10 and TGF-β, as well as through direct cell-cell interactions, thereby ultimately suppressing anti-tumor immunity (195). Notably, IL-10-producing Bregs (B10 cells) are significantly expanded in the peripheral blood of ESCC patients (196). Mao et al. demonstrated that ESCC-derived exosomes drive B10 differentiation and IL-10 secretion, suppressing effector immune cells while expanding both Bregs and Tregs, thereby establishing an exosome-mediated positive feedback loop (100). Beyond immunosuppression, B cells can promote tumor cell proliferation and migration through secretion of angiogenic factors such as VEGF, fostering paracrine interactions that drive EC neovascularization (192). In ESCC, cancer-derived high-mobility group box 1 (HMGB1) enhances B cell proliferation and migration, inducing a pro-angiogenic phenotype characterized by VEGF production (197). Thus, Bregs and tumor-educated B cells not only suppress cytotoxic immunity but also actively remodel the TME into a pro-angiogenic, immune-excluded niche, thereby consolidating resistance to ICI therapy.

Collectively, Tregs and Bregs constitute a collaborative immunosuppressive axis in the TME of EC rather than isolated barriers. Through reciprocal amplification via shared cytokine networks and direct cellular cross-talk, these regulatory lymphocytes collectively suppress cytotoxic T, NK, and B-cell responses while promoting angiogenesis and myeloid cell recruitment (195).

#### 3.3.4 NK-cell dysfunction and loss of innate immune surveillance

NK cells, as a critical component of the innate immune system, have an important role in defending the body against malignant tumors (198). In EC, they are recruited to the tumor site by pro-inflammatory chemokines secreted by innate and adaptive immune cells (57). Upon entering the TME, NK cells recognize malignant cells through the “missing-self” mechanism: reduced MHC-I expression on tumor cells relieves inhibitory signaling via killer-cell immunoglobulin-like receptors (KIRs), triggering cytotoxic degranulation (198). This renders NK cells particularly effective against tumors that evade T-cell recognition through MHC-I downregulation. Beyond direct lysis, NK cells secrete IFN-γ and TNF-α, exerting anti-proliferative, anti-angiogenic, and pro-apoptotic effects (199, 200). However, the immunosuppressive TME severely impairs NK-cell function. In this regard, tumors shed soluble MICA and MICB that bind NKG2D, blocking its activation and receptor expression on NK cells (201). Recently, SAMD3^+^ NK cells were identified as a dysfunctional subset within tumors; these cells exhibit attenuated cytotoxicity, interact with exhausted CD8^+^ T cells, and compromise chemotherapeutic efficacy in EC. Notably, SAMD3 knockdown restores NK-cell cytotoxicity and restrains tumor growth, identifying SAMD3 as a tractable target to reverse innate immune escape (202). Elucidating the mechanisms that govern NK-cell dysfunction in the EC microenvironment is therefore essential for restoring innate surveillance and advancing NK-cell-directed immunotherapies (203).

Lymphocyte-mediated immune resistance in EC arises from coordinated defects in adaptive and innate immunity. Impaired CD8^+^ T-cell recruitment and activation, driven by epigenetic silencing and checkpoint signals, cripple effector infiltration. Concomitantly, Treg/Breg immunosuppression, terminal T-cell exhaustion, and NK-cell dysfunction collectively sustain tumor persistence and ICI failure. Preserving Tpex, disrupting Breg/Treg networks, and restoring NK effector functions may represent rational avenues to relieve these interconnected barriers and improve immunotherapeutic outcomes in EC.

### 3.4 Stromal and vascular remodeling in immune microenvironment regulation

While myeloid and lymphoid populations actively suppress immune function through soluble mediators and checkpoint interactions, stromal and vascular components impose a distinct layer of resistance by physically restructuring the TME. In EC, CAFs, tumor-associated endothelial cells (TAECs), and cancer stem cells (CSCs) collectively establish dense ECM barriers, alter tissue mechanics, and generate hypoxic niches that limit both immune surveillance and therapeutic access (123).

#### 3.4.1 CAFs and immune exclusion

CAFs represent the most abundant stromal population in the EC microenvironment, arising from normal fibroblasts upon activation by tumor-derived TGF-β and exosomal signals (16). Rather than forming a uniform population, CAFs exhibit marked functional heterogeneity that directly determines whether T cells can access malignant nests. Single-cell transcriptomic profiling has resolved this complexity into distinct functional states (88), providing a framework to understand how specific CAF subsets differentially regulate immune exclusion.

Broadly, three interrelated CAF subpopulations orchestrate this process in EC. Inflammatory CAFs (iCAFs) are characterized by robust secretion of IL-6 and CXCL12 (101, 102, 204). Beyond recruiting immunosuppressive myeloid cells into the stroma, iCAF-derived IL-6 cooperates with exosomal miR-21 to activate STAT3 signaling, which drives the generation of monocytic MDSCs and paralyzes DC maturation, effectively disabling anti-tumor immunity before T cells encounter tumor antigens (114). By contrast, myofibroblastic CAFs (myCAFs) display prominent TGF-β signaling and high α-smooth muscle actin (α-SMA) expression (205). These cells are chiefly responsible for the deposition and cross-linking of collagenous ECM, generating a dense, stiffened matrix that acts as a physical barrier to both drug penetration and immune-cell infiltration (206). TGF-β further exacerbates immune exclusion by upregulating laminin γ2 (Ln-γ2) in ESCC cells through the JNK/AP1 pathway, thereby repelling T cells from the tumor parenchyma and driving resistance to anti-PD-1 therapy (207, 208). Another population, antigen-presenting CAFs (apCAFs), expresses MHC class II-related molecules yet generally lacks adequate co-stimulatory signals (88). In EC, this incomplete antigen presentation may result in ineffective CD4^+^ T-cell priming or even diversion toward regulatory phenotypes, further dampening cytotoxic responses (209).

Several additional CAF-derived axes reinforce this exclusionary landscape. In primary ESCC, CAF-secreted WNT2 impairs DC differentiation and subsequent T-cell activation through the SOCS3/p-JAK2/p-STAT3 pathway; notably, WNT2 blockade restores DC-mediated immunity and sensitizes tumors to PD-1 inhibition (210). The co-inhibitory TIGIT/NECTIN2 axis between specific CAF subsets and Tregs further entrenches an immunosuppressive milieu in ESCC (211). Collectively, these heterogeneous CAF states link inflammatory signaling, defective DC maturation, myeloid-cell reprogramming, TGF-β activity, and ECM remodeling to T-cell exclusion and immunotherapy resistance. Therapeutic strategies that selectively disrupt immunosuppressive CAF states or their downstream signaling and matrix-remodeling programs may therefore enhance anti-PD-1/PD-L1 efficacy in EC.

#### 3.4.2 ECM stiffness and mechanotransduction

Beyond secreted mediators, CAFs deposit and cross-link collagen to generate a stiffened ECM that physically excludes immune cells and impedes drug delivery. Progressive collagen accumulation increases matrix rigidity, obstructing CD8^+^ T-cell migration and limiting therapeutic antibody penetration into deep tumor regions (18, 206). This mechanical microenvironment is actively sensed through integrin-focal adhesion kinase (FAK) signaling, which activates Yes-associated protein (YAP)-dependent transcriptional programs that perpetuate fibroblast activation and further amplify collagen deposition (212). Consequently, the stiffened matrix not only forms a passive physical barrier but also functionally suppresses lymphocyte infiltration and compromises the distribution of both chemotherapeutics and antibody-based immunotherapeutic agents (213, 214). Collectively, integrin-FAK-YAP-mediated mechanotransduction and ECM stiffness constitute a structural layer of immune exclusion that cooperates with CAF-driven immunosuppression to reinforce therapeutic resistance in EC.

#### 3.4.3 Abnormal angiogenesis and TAECs

TAECs constitute another critical stromal constituent that shapes the immunosuppressive TME through vascular dysregulation (215, 216). Under hypoxic conditions, these cells acquire stem-like features and drive the formation of neo-vessels that are characteristically leaky, tortuous, and poorly perfused (216, 217). In ESCC, cancer-derived exosomal miR-21 enters TAECs, targets PTEN, and activates the Akt pathway, thereby stimulating endothelial proliferation and migration (218). Concurrently, Notch-driven upregulation of USP5 stabilizes STAT3, promoting the secretion of VEGF, ANGPT2, and CXCL1. These factors generate structurally abnormal vasculature that physically impedes immune-cell infiltration while fostering immune evasion (219). The resulting vascular dysfunction establishes a self-reinforcing hypoxia-angiogenesis feedback loop, wherein chronic hypoxia further amplifies pro-angiogenic signaling and perpetuates vascular abnormality (220).

Functionally, this aberrant endothelium actively restricts immune surveillance by altering the expression profile of molecules required for leukocyte trafficking, thereby impairing T-cell adhesion and transendothelial migration (215, 221). GPR87 activation in EC similarly drives STAT3-dependent VEGFA upregulation, stimulating pathological angiogenesis and consolidating an immunosuppressive microenvironment (222). Elevated IL-33 expression in ESCC microvascular TAECs further marks highly proliferative populations associated with lymph node metastasis and poor overall survival (223). Together, these observations indicate that TAEC-mediated vascular remodeling is not merely a passive consequence of tumor growth but an active determinant of immune exclusion, supporting the clinical evaluation of anti-angiogenic combinations in EC.

#### 3.4.4 CSC-stromal interactions and resistant niches

Within the structurally remodeled TME, CSCs exploit stromal signals to establish niches that are refractory to immune attack and therapeutic elimination (224). CSCs are characterized by stem-like properties (225, 226), including self-renewal and multipotent differentiation capacity, and their maintenance in EC is heavily dependent on microenvironmental cues such as hypoxia, abnormal angiogenesis, and chronic inflammation (227, 228). In ESCC, stromal-derived signals including TGF-β and IL-6 sustain CSC stemness and promote EMT-associated plasticity, enabling tumor cells to acquire invasive and drug-resistant phenotypes (229).

A particularly clinically relevant subset comprises dormant cancer stem cells (DCSCs) (230, 231), which evade immune clearance through robust PD-L1 expression and physically exclude T cells via QSOX1 upregulation, thereby establishing a self-reinforcing resistant niche that sustains both chemoresistance and metastatic potential (232). In EAC, CSCs further exhibit elevated expression of ion channel and transporter genes, including transient receptor potential vanillin subtype 2 (TRPV2) and solute carrier family 12 member 2 (SLC12A2), which contribute to therapeutic resistance (233). By integrating CAF-derived pro-survival signals, EMT programs, and immune-checkpoint upregulation, CSCs function as persistent cellular reservoirs that drive tumor recurrence and acquired immunotherapy resistance. Disrupting these CSC-stromal interactions therefore represents a promising strategy to overcome treatment failure in EC.

### 3.5 Metabolic dysregulation and immune suppression

Metabolic dysregulation is not a parallel feature but a central mechanism of immune resistance in EC (**Figure 4**) (15). Rapidly proliferating tumor cells compete with infiltrating immune cells for nutrients and generate metabolites that directly impair immune-cell activation (234), while suppressive myeloid and stromal cells undergo metabolic adaptations that reinforce their immunoregulatory phenotypes (15). These processes intersect with hypoxia, oxidative stress, acidosis, and mitochondrial dysfunction to create a hostile metabolic niche that drives lymphocyte dysfunction and immunotherapy resistance.

**Figure 4:**
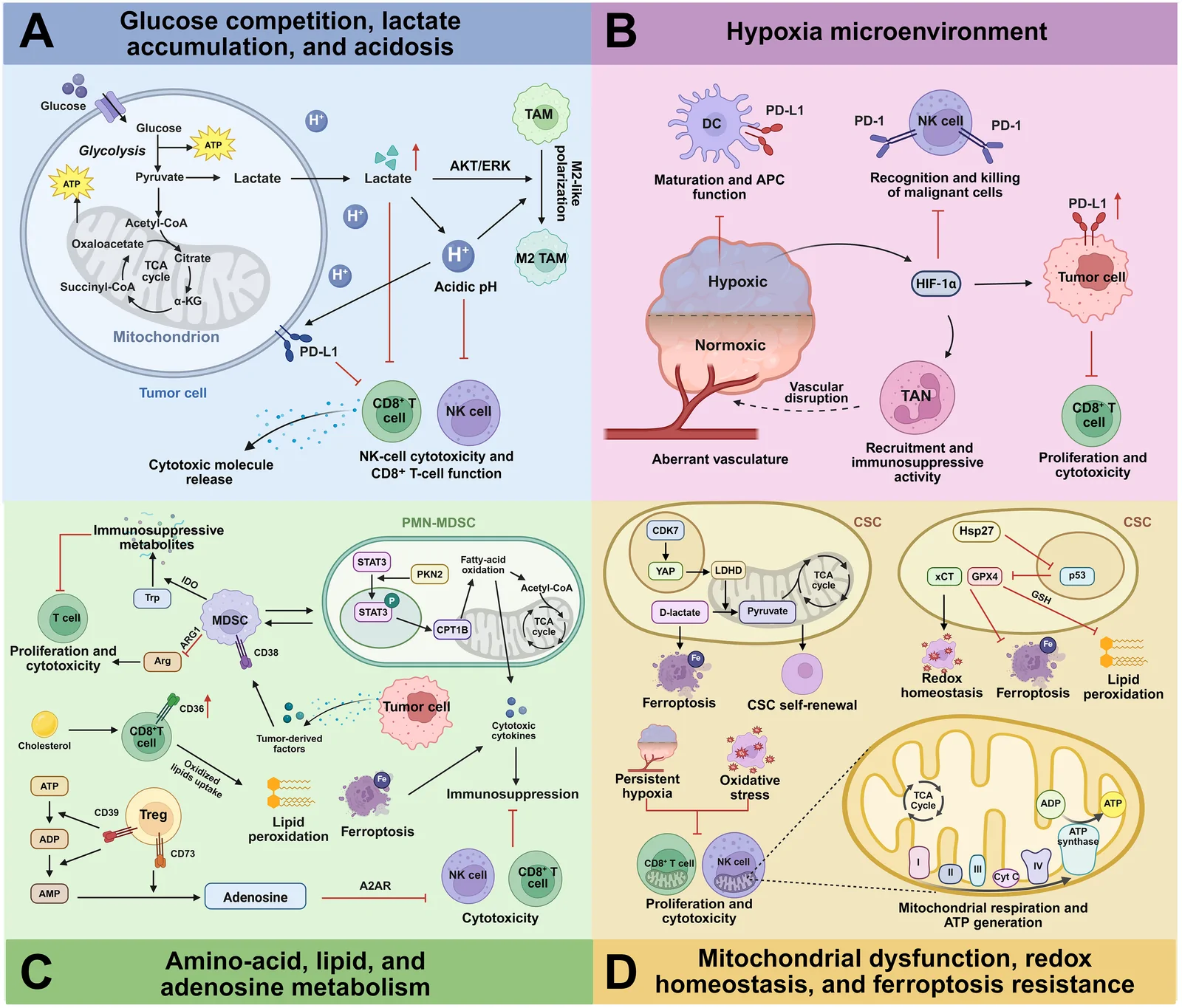
Metabolic dysregulation drives immunosuppression and immunotherapy resistance in EC. **(A)** Tumor glycolysis depletes glucose and generates lactate and H^+^, promoting M2-like TAM polarization via AKT/ERK and suppressing CD8^+^ T-cell and NK-cell cytotoxicity under acidic pH. **(B)** Aberrant vasculature creates hypoxia, stabilizing HIF-1α to upregulate tumor PD-L1, recruit immunosuppressive TANs, impair DC maturation and APC function, and suppress NK-cell and CD8^+^ T-cell activity. **(C)** MDSCs suppress T cells via ARG1-mediated arginine depletion and IDO-driven tryptophan catabolism. The ectonucleotidases CD39/CD73 generate adenosine, which inhibits effector cells through A2AR. CD38^+^ MDSCs enhance suppression via NF-κB-driven iNOS. Elevated cholesterol upregulates CD36 on CD8^+^ T cells, promoting lipid peroxidation and ferroptosis. In PMN-MDSCs, the PKN2-STAT3-CPT1B axis drives fatty-acid oxidation to sustain immunosuppression. **(D)** CSCs resist ferroptosis through the CDK7-YAP-LDHD axis and the Hsp27-GPX4 pathway. Concurrently, persistent hypoxia and oxidative stress impair mitochondrial respiration and ATP generation in CD8^+^ T cells and NK cells, crippling their cytotoxic capacity. A2AR, A2A receptor; APC, antigen-presenting cell; ARG1, arginase 1; CDK7, cyclin-dependent kinase 7; CPT1B, carnitine palmitoyltransferase 1B; CSC, cancer stem cell; DC, dendritic cell; EC, esophageal carcinoma; GPX4, glutathione peroxidase 4; HIF-1α, hypoxia-inducible factor-1α; Hsp27, heat shock protein 27; IDO, indoleamine 2, 3-dioxygenase; LDHD, D-lactate dehydrogenase; MDSC, myeloid-derived suppressor cell; NF-κB, nuclear factor kappa B; NK, natural killer; PD-L1, programmed death-ligand 1; PMN-MDSC, polymorphonuclear myeloid-derived suppressor cell; PKN2, protein kinase N2; STAT3, signal transducer and activator of transcription 3; TAM, tumor-associated macrophage; TAN, tumor-associated neutrophil; YAP, Yes-associated protein.

#### 3.5.1 Glucose competition, lactate accumulation, and acidosis

Rapidly proliferating EC cells frequently rely on aerobic glycolysis to meet energetic demands, resulting in increased glucose consumption and lactate production that create a nutrient-depleted and acidic microenvironment (235). Competition for glucose restricts the metabolic fitness and effector function of tumor-infiltrating CD8^+^ T cells and NK cells (236, 237), thereby weakening anti-tumor immunity. Lactate, a major product of glycolytic metabolism (238), further reinforces suppression through multiple mechanisms (239): it impairs NK-cell cytotoxicity and CD8^+^ T-cell function while promoting TAM polarization toward an M2-like immunosuppressive state (240, 241). Zhang et al. demonstrated that lactate secreted by EC cells induces M2 TAM polarization via the AKT/ERK signaling pathway, promoting EC cell migration and tumor growth (242). Concurrent extracellular acidification exacerbates immunosuppression by increasing PD-L1 expression (243) and interfering with cytotoxic molecule release from T and NK cells (244). Together, glucose depletion, lactate accumulation, and acidosis form an interconnected metabolic barrier that suppresses effector lymphocytes and contributes to immunotherapy resistance.

#### 3.5.2 Hypoxia microenvironment

Hypoxia is another major metabolic and structural feature of the EC microenvironment. Rapid tumor growth, insufficient oxygen delivery, and abnormal vasculature generate regions of persistent hypoxia (245), which alters the phenotype and function of tumor cells, myeloid cells, lymphocytes, and stromal cells to establish a self-reinforcing immunosuppressive niche (246). Hypoxia-inducible factor-1 alpha (HIF-1α) serves as the central mediator of hypoxia-driven immune dysregulation (247, 248). It upregulates PD-L1 expression in tumor cells, limiting CTL activation and facilitating immune escape (249). Hypoxia also promotes neutrophil recruitment and immunosuppressive activity. Because neutrophils possess high glycolytic capacity, they survive under low-oxygen conditions and further aggravate local hypoxia by damaging tumor vasculature, creating a positive-feedback loop that reinforces HIF signaling (250, 251). Hypoxia directly impairs anti-tumor immunity by suppressing NK-cell activity through downregulation of activating receptors and granzyme B degradation (252, 253), impairing DC maturation and antigen presentation (254), and limiting CD8^+^ T-cell proliferation, cytokine production, and cytotoxic function (255). Therapeutic strategies aimed at normalizing tumor vasculature, improving oxygen delivery, or inhibiting HIF-dependent signaling may therefore enhance anti-tumor immunity in EC (256).

#### 3.5.3 Amino-acid, lipid, and adenosine metabolism

Beyond glucose and oxygen deprivation, altered amino-acid, lipid, and purine metabolism substantially contributes to immune suppression in the EC microenvironment. Among these pathways, amino-acid metabolism promotes immune suppression primarily through arginine and tryptophan depletion (257). MDSCs deplete arginine via ARG1, reducing T-cell receptor CD3ζ expression and impairing T-cell proliferation and antigen-specific responses (258), while MDSC-derived iNOS and ROS further disrupt T-cell signaling (259). Tumor cells and other suppressive populations additionally express indoleamine 2, 3-dioxygenase (IDO), which catabolizes tryptophan into immunosuppressive metabolites that impair effector T-cell proliferation and cytotoxicity. In ESCC, tumor-cell IDO expression is associated with reduced CD8^+^ TILs and poorer overall survival (260).

Lipid metabolism also drives functional reprogramming of suppressive cells (261). In PMN-MDSCs, protein kinase N2 (PKN2) promotes STAT3 phosphorylation and CPT1B transcription, thereby enhancing CPT1B-mediated fatty-acid oxidation that supports their survival and immunosuppressive activity in the nutrient-poor TME (262). Elevated tumor-microenvironmental cholesterol upregulates CD36 on tumor-infiltrating CD8^+^ T cells (263), and CD36-mediated uptake of oxidized lipids drives lipid peroxidation and ferroptosis (264), reducing cytotoxic cytokine production and impairing anti-tumor function (263).

Adenosine-mediated purinergic signaling further suppresses effector lymphocytes. Ectonucleotidases expressed by tumor cells and immune cells can convert extracellular nucleotides into adenosine (265). CD39 contributes to the initial hydrolysis of extracellular ATP and ADP (266), while downstream ectonucleotidase activity generates immunosuppressive adenosine that dampens the CD8^+^ T-cell and NK-cell cytotoxicity (187, 188, 267). Downstream A2A receptor engagement further suppresses MAPK signaling while upregulating TGF-β, FOXP3, and immune checkpoints (PD-1, CTLA-4, LAG-3), thereby facilitating tumor escape (188). CD38, another ectoenzyme involved in extracellular nucleotide metabolism and immune regulation, is highly expressed on MDSCs (115). CD38^+^ MDSCs display a more immature and immunosuppressive phenotype, with stronger T-cell-suppressive activity and greater tumor-promoting capacity than their CD38^-^ counterparts (116). Tumor-derived factors, including IL-6, IGFBP-3, CXCL16, IFN-γ, and TNF-α, can induce CD38 expression, while downstream NF-κB activation enhances iNOS production and further reinforces immunosuppression (116). Together, amino-acid depletion, fatty-acid oxidation, and adenosine accumulation represent complementary metabolic mechanisms through which suppressive cells converge on T-cell and NK-cell dysfunction.

#### 3.5.4 Mitochondrial dysfunction, redox homeostasis, and ferroptosis resistance

Mitochondrial function and redox balance are closely linked to tumor persistence and therapeutic resistance in EC (268–270). Reprogrammed cellular metabolism is particularly important for maintaining CSCs (271). CDK7 enhances YAP transcriptional activity to promote D-lactate dehydrogenase (LDHD) expression (272), which is significantly higher in CSCs than in differentiated tumor cells and is associated with poor prognosis in ESCC (273). The CDK7-YAP-LDHD axis supports CSC self-renewal by facilitating D-lactate elimination and thereby conferring ferroptosis resistance (273). ESCC stem-like cells additionally resist ferroptotic cell death through activation of the Hsp27-GPX4 pathway, which maintains glutathione-dependent redox homeostasis and limits lipid peroxidation (274). These adaptations enable tumor cells to survive under oxidative and metabolic stress.

Mitochondrial dysfunction exerts divergent effects across cellular compartments. Persistent hypoxia, nutrient deprivation, oxidative stress, and suppressive metabolites impair mitochondrial respiration and ATP generation in T and NK cells (255, 275), limiting their proliferation and cytotoxicity. By contrast, tumor cells and CSCs adapt their mitochondrial and lipid metabolism to sustain redox homeostasis and survival in the same hostile environment (271–274). Targeting mitochondrial metabolism, ferroptosis resistance, or redox-regulatory pathways may therefore simultaneously weaken metabolically adaptable tumor cells and restore anti-tumor immune function.

Collectively, the metabolic programs described above are not isolated features of the TME of EC. Instead, they form an integrated network in which tumor, myeloid, and stromal cells converge to deprive effector lymphocytes of nutrients and impair their cytotoxic function, thereby driving both primary and acquired resistance to immunotherapy.

### 3.6 Crosstalk-driven primary and acquired resistance

Primary resistance is frequently predetermined by the baseline TME architecture before ICI exposure. Tumors presenting an immune-desert phenotype fail to recruit CD8^+^ cytotoxic T cells from the outset (166, 171). Conversely, immune-excluded tumors harbor Teffs at the invasive margin but are physically barred from the tumor parenchyma by CAF-secreted TGF-β, laminin-γ2, and dense collagen deposition (207, 276). A pre-existing myeloid-rich microenvironment dominated by PD-L1^+^ MDSCs and M2-polarized TAMs further poisons the niche through arginine depletion, ROS generation, and PD-1/PD-L1-mediated paralysis of infiltrating lymphocytes (111, 116, 122). Concurrently, hypoxic and acidic metabolic conditions—driven by tumor glycolysis and aberrant vasculature—impair T-cell and NK-cell fitness while enforcing terminal exhaustion epigenetically, rendering the tumor refractory to checkpoint blockade before treatment begins (167, 240).

Acquired resistance evolves dynamically during ICI therapy. Sustained IFN-γ signaling from initially activated T cells triggers adaptive PD-L1 upregulation on both tumor cells and TAMs, establishing a feedback loop that exhausts nascent anti-tumor immunity (131). Under this selective pressure, tumor subclones with defective antigen-presentation machinery emerge and evade T-cell recognition (277). The TME concurrently undergoes compensatory myeloid expansion: MDSCs and M2-TAMs are recruited via IL-6, CCL2, and CSF-1 signaling, replenishing the immunosuppressive pool and outcompeting effector cells for nutrients and oxygen (114, 124, 129). Within the lymphoid compartment, the finite Tpex reservoir is progressively depleted and differentiates into terminally exhausted Tex cells marked by TOX and CD39, which are epigenetically fixed and unresponsive to PD-1 blockade (92, 93). Therapy-induced stromal remodeling further consolidates resistance: CAFs upregulate IL-6, CCL2, and ECM proteins that physically exclude T cells and limit drug penetration, while abnormal angiogenesis perpetuates hypoxia (211, 220).

These mechanisms also operate at different stages of treatment. Pre-existing myeloid enrichment, defective antigen presentation, dense ECM, abnormal vasculature, and metabolically hostile niches may prevent an initial response and thereby promote primary resistance. In contrast, continued antigen exposure, adaptive checkpoint upregulation, compensatory recruitment of suppressive cells, and therapy-induced remodeling of stromal and metabolic niches can promote acquired resistance after an initial response. Therefore, effective strategies to overcome resistance in EC must simultaneously dismantle multiple, co-evolving TME barriers rather than targeting isolated cell populations or single signaling axes.

### 3.7 The heterogeneity of EC TME and response to immunotherapy

The TME of EC exhibits profound inter-tumoral and intra-tumoral heterogeneity spanning spatial architecture, temporal evolution, cellular composition, and molecular states. This heterogeneity constitutes a fundamental basis for variable ICI efficacy and the emergence of both primary and acquired resistance (228, 278). Recent advances in single-cell and spatial omics have begun to deconvolute this complexity, revealing that effective immunotherapy requires moving beyond broad histological classifications toward mechanism-based, microenvironment-guided stratification (88, 211).

#### 3.7.1 Spatial immune phenotypes and TLS

Immunophenotyping based on CD3^+^ and CD8^+^ T-cell density and localization delineates EC into at least four distinct categories: immune-desert, immune-excluded, inflamed, and immunosuppressed subtypes, each associated with differential responses to ICI therapy (278). In ESCC, spatial transcriptomics has revealed a highly organized yet heterogeneous architecture. The invasive tumor front is characterized by a CAF-epithelial niche driven by JAG1/NOTCH1 signaling, which establishes a physical and biochemical barrier that impedes CD8^+^ T-cell infiltration, corresponding to an immune-excluded phenotype (228). Conversely, the tumor core often harbors exhausted CD8^+^ T cells and M2-polarized TAMs, whereas approximately 30% of cases display TLS in peritumoral regions associated with activated immune states (279–281). High densities of mature TLS containing proliferative CD20^+^Ki-67^+^ B cells, CD21^+^ DCs, and activated CD8^+^ CTLs are robust predictors of favorable survival and enhanced responses to neoadjuvant chemoradiotherapy combined with immunotherapy (282, 283). Notably, the spatial context matters: intra-tumoral and invasive-margin TLS are generally associated with good prognosis, whereas abundant peritumoral TLS can correlate with poorer outcomes, possibly reflecting compensatory immune organization at the tumor periphery (283). Spatial dynamics of CD39^+^CD8^+^ exhausted T cells further reveal that TLS-mediated response to PD-1 blockade is orchestrated through localized T-cell reactivation within these structures, underscoring that TLS are active participants in anti-tumor immunity (92).

#### 3.7.2 Temporal heterogeneity and therapy-induced remodeling

The EC TME undergoes continuous dynamic evolution from precancerous lesions to invasive carcinoma and is further reshaped by therapeutic intervention. During carcinogenesis, the microenvironment transitions from an immune-clearance state toward an increasingly immunosuppressive milieu characterized by expanding Tregs, SPP1^+^ macrophages, and CAF-mediated ECM deposition (228, 284). Neoadjuvant immunochemotherapy offers a critical window to observe this plasticity: responders typically exhibit post-treatment increases in M1-like TAMs, mature DCs, and CD8^+^ Tpex subsets, accompanied by enhanced IFN-γ signaling (93, 94). In contrast, non-responders retain or amplify immunosuppressive features, including enrichment of LRRC15^+^ CAFs, SPP1^+^ macrophages, and Tex, suggesting that pre-existing stromal and myeloid niches constrain therapeutic reprogramming (94, 285). These findings establish that the TME should be understood as a temporally evolving ecosystem rather than a fixed background.

#### 3.7.3 ESCC versus EAC: distinct immune landscapes

Although often discussed under the umbrella of EC, ESCC and EAC display markedly different molecular characteristics and immune microenvironments that influence therapeutic strategy. ESCC typically exhibits a higher tumor mutational burden (TMB), more frequent copy-number alterations, and greater infiltration of cytotoxic lymphocytes, which may underlie its relatively higher ORR to PD-1/PD-L1 blockade (57, 286). In contrast, EAC frequently arises in the context of Barrett’s esophagus, displays distinct driver mutation profiles (e.g., TP53, CDKN2A, SMAD4), and is often dominated by a desmoplastic stroma with abundant myCAFs and altered angiogenic signaling (286, 287). These histology-specific differences necessitate tailored immunotherapeutic approaches. For instance, targets such as CLDN18.2 are relevant primarily in EAC and gastroesophageal junction adenocarcinoma (288, 289), whereas ESCC may benefit more from strategies addressing myeloid-rich inflammation and ECM-mediated immune exclusion (290, 291).

#### 3.7.4 Bridging heterogeneity to therapy

Confronting TME heterogeneity demands a shift from empirical combination regimens to mechanism-guided, phenotype-specific interventions. For immune-desert tumors, strategies that promote initial T-cell priming may convert cold lesions into inflamed phenotypes; for immune-excluded tumors, targeting myCAFs or normalizing collagen architecture may restore T-cell access; for inflamed yet immunosuppressed tumors, combining PD-1/PD-L1 blockade with myeloid-targeting or metabolic-modulating agents represents a rational approach to dismantle suppressive niches. These immunosubtypes may inform patient selection for ICI-based combination therapy.

## 4 Therapeutic strategies to overcome immunotherapy resistance

Immunotherapies dominated by ICI still face numerous challenges, such as low treatment response rates, drug resistance, and potential systemic adverse effects (292). Therefore, it is important to understand their immunological mechanisms and explore novel approaches to enhance the efficacy of immunotherapies. Notably, remodeling TME is another practical way to improve the efficacy of ICIs and overcome immunotherapy resistance. Multiple research teams have been dedicated to exploring and developing novel therapeutic strategies targeting immunotherapy resistance (**Figure 5**).

**Figure 5:**
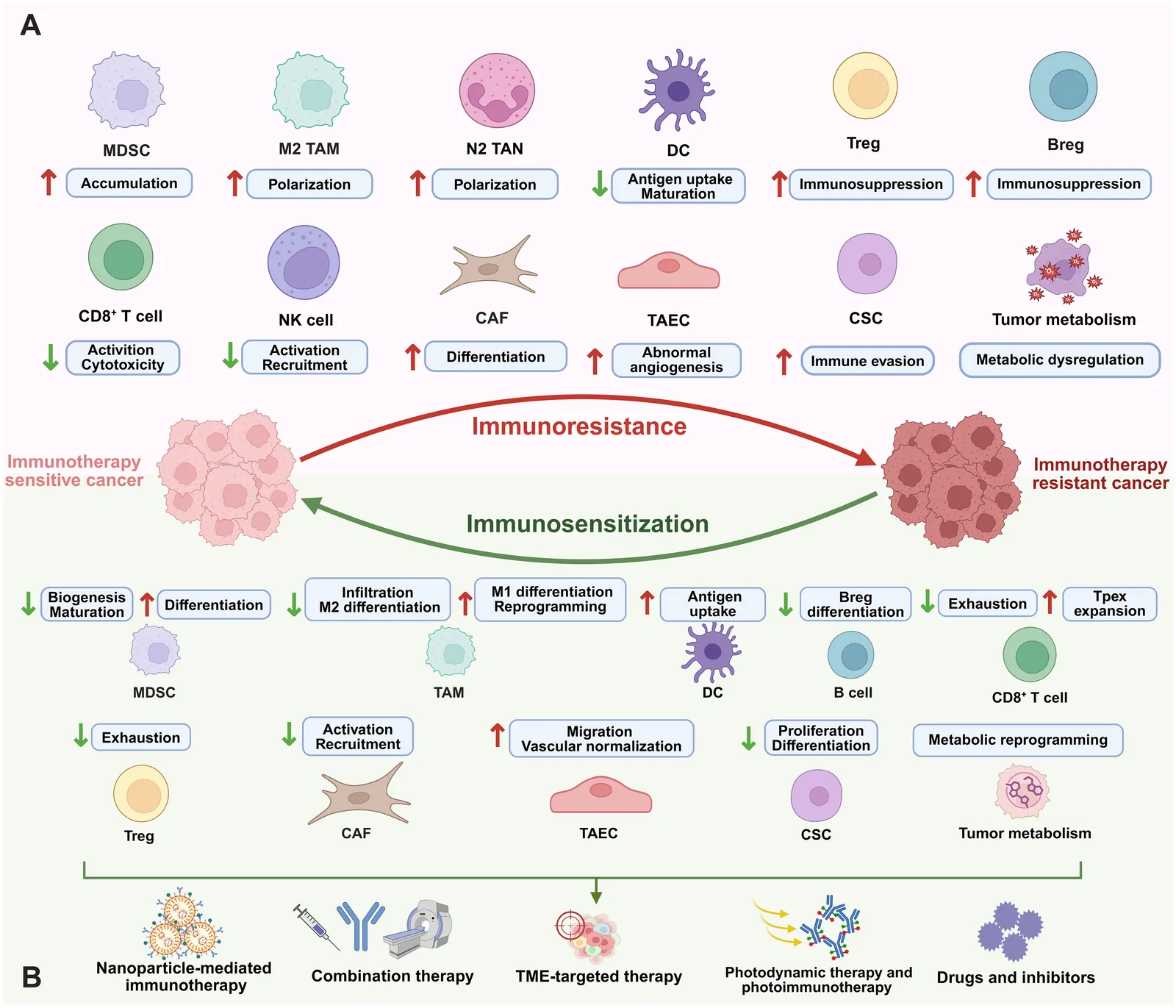
TME-mediated immune tolerance and therapeutic sensitization in EC. **(A)** Diverse TME components undergo functional reprogramming that drives immunotherapy resistance. **(B)** Therapeutic strategies to overcome immunotherapy resistance. TME, tumor microenvironment; EC, esophageal carcinoma; MDSC, myeloid-derived suppressor cell; M2 TAM, M2-polarized tumor-associated macrophage; N2 TAN, N2-polarized tumor-associated neutrophil; DC, dendritic cell; NK cell, natural killer cell; Breg, regulatory B cell; CD8^+^, cluster of differentiation 8; Treg, regulatory T cell; CAF, cancer-associated fibroblast; TAEC, tumor-associated endothelial cell; CSC, cancer stem cell.

### 4.1 Targeting myeloid cells in the TME of EC

#### 4.1.1 Targeting MDSCs

Studies have shown that targeted regulation of MDSC biosynthesis can reduce the burden of MDSCs and enhance the efficacy of immunotherapy (293). For EC resistant to ICI therapy, targeting specific high-expressing cytokines in MDSCs can enhance immunotherapy efficacy (292). Qin et al. revealed that GPR84 exhibits significantly high expression on MDSCs in clinical samples of EC and murine tumor models. In EC patients resistant to anti-PD-1 therapy, GPR84^+^ MDSCs and PD-L1^+^ MDSCs are markedly clustered, and high GPR84 expression is a negative factor influencing OS following anti-PD-1 treatment. Studies have also demonstrated that the combination of GPR84 antagonists with anti-PD-1 antibodies enhances anti-tumor efficacy in ESCC mice (293). Moreover, tRF-22 regulates TGF-β2 expression, and its accumulation promotes MDSC generation, thereby driving the immunosuppressive process in ESCC patients. The combination of a tRF-22 antagonist or a TGF-β signaling inhibitor with anti-PD-1 therapy enhances immune responses and inhibits tumor growth (294). Another approach achieves therapeutic effects by influencing the aggregation and function of MDSCs. Eva-1 Homolog B (EVA1B) expression is significantly upregulated in ESCC patient tissues and correlates with EC progression stages and increased abundance of MDSCs. Targeted inhibition of EVA1B decreased the expression of EMT pathway-related proteins (e.g., Wnt3a, β-catenin, LRP6), and effectively suppressed the expansion and recruitment of MDSCs in the immune microenvironment of mouse models (82). Although the role of MDSCs in promoting tumor growth and suppressing immune responses has been extensively investigated, effective clinical targeting strategies remain lacking and need further validation.

#### 4.1.2 Targeting TAMs

Therapeutic strategies targeting TAMs are being actively explored, including reducing macrophage infiltration, inhibiting M2 polarization, promoting M1 repolarization, and inducing phenotypic conversion (85). In ESCC, SPP1 recruits macrophages and promotes M2 polarization, inducing VEGFA and IL-6 secretion; RNA aptamer-mediated SPP1 blockade significantly inhibits tumor growth and M2-TAM infiltration (295). The FOXF2/RNF144A/FTO axis also regulates TAM polarization, whereby FOXF2 overexpression promotes FTO ubiquitination and degradation, thereby suppressing M2 polarization (296). Additionally, creatine accumulation coupled with HK3 deficiency drives M2-like TAM polarization through metabolic remodeling, and targeting this axis reverses immunosuppression and enhances immunotherapy efficacy (297). Promoting M1-like repolarization represents another viable strategy. P-Hydroxylcinnamaldehyde induces M1 polarization via NF-κB and Toll-like receptor pathways, and increases immune-activating proteins, thereby suppressing tumor growth in ESCC mouse models (298). Moreover, sitravatinib, a multi-target TKI targeting TAM receptors, promotes M2-to-M1 transition and enhances CD8^+^ T-cell infiltration when combined with PD-1 blockade in EAC (299). Treatment-induced immunosuppressive TAMs also offer therapeutic targets. Radiotherapy induces DYNLL1-AS1-enriched extracellular vesicles from ESCC cells, reprogramming macrophages into PD-L1^+^ TAMs. Targeting DYNLL1-AS1 combined with PD-L1 blockade may reverse this effect (300). Besides, tumor-intrinsic cGAS-STING signaling promotes M2-TAM recruitment via IL-34 expression, and blocking IL-34 enhances radiotherapy combined with ICIs (301). Similarly, neoadjuvant chemotherapy upregulates IL-34, increasing CD163^+^ TAMs and fostering chemoresistance. Blocking IL-34 signaling to inhibit M2-TAM polarization thus offers a strategy to overcome chemoresistance in ESCC patients (302). Therefore, investigating these novel molecular characteristics of TAMs facilitates identification of clinically valuable targets associated with immunosuppression in EC.

#### 4.1.3 Targeting DCs

Since many tumor antigens are self-antigens, DCs induce immune tolerance and resistance to immunotherapy (303). DC-based immunotherapies aim to overcome this resistance by restoring DC function to trigger an effective anti-tumor immune response (304). Three DC subtypes were identified, with pDCs and tDCs linked to prognosis. Targeting these DC subsets could improve ESCC patient stratification and guide immunotherapy, offering new therapeutic avenues (305). A novel vaccine uses an anti-CLEC9A antibody to deliver the NY-ESO-1 antigen specifically to human CD141^+^ DCs (cDC1 subset). This targeting enhances cross-presentation and activates NY-ESO-1-specific CD8^+^ T cells, inducing potent anti-tumor immunity. It outperforms non-targeted or DEC-205-targeted vaccines, effectively priming naïve T cells with tumor-killing capacity (306). Radiotherapy upregulates TIGIT expression within the EC microenvironment. TIGIT blockade enhances tumor response to radiotherapy via a CD103^+^ DC-dependent mechanism, thereby potentiating CD8^+^ T cell-mediated anti-tumor immunity. CD103^+^ DCs accumulate following treatment and are essential for T cell activation. Moreover, Flt3L therapy further improves efficacy by expanding the CD103^+^ DC compartment (307). Integrating these DC-centered modalities could synergistically refine patient selection and unlock durable, curative immunity in the clinical care of EC.

### 4.2 Targeting lymphoid cells in the TME of EC

#### 4.2.1 Targeting B cells

Accumulating evidence has revealed a dualistic and context-dependent role for B cells in the TME of EC (308). While certain subsets are associated with favorable prognosis and robust responses to ICI therapy, others drive chronic inflammation, immunosuppression, and therapeutic resistance (309). ESCC is heavily infiltrated by B cells and plasma cells, with notable clonal expansion and IgG subclass switching (310). Higher levels of immunoglobulins, CD138, and IgG2-producing plasma cells strongly correlate with improved survival, highlighting protective B-cell populations as promising biomarkers and supporting developing B cell-targeted immunotherapies for ESCC (311). In advanced ESCC, increased B-cell signatures, naïve B cells, plasma cells, and stromal CD19 expression are linked to prolonged PFS and OS in patients receiving anti-PD-1/PD-L1 therapy (312). In treatment-naïve ESCC, TLSs are associated with favorable prognosis and are enriched for IRF4-expressing B cells. Mechanistically, competitive binding of CD40 and STING with TRAF2 promotes IRF4 through non-canonical NF-κB signaling, thereby driving IRF4-mediated B cell activation (313). Conversely, B-cell infiltration declines during ESCC progression but is enriched in immunotherapy-resistant patients. Resistance is mediated by cholesterol-biosynthetic tumor cells expressing macrophage migration inhibitory factor (MIF), which disrupts germinal center B-cell responses via MIF-CXCR4 signaling and impairs humoral immunity. Targeting this crosstalk may restore B-cell-mediated anti-tumor effects in ESCC (314). Pathogenic B-cell subsets also promote immunosuppression and treatment resistance. Senescent EGR1^+^ B cells drive chronic inflammation via the senescence-associated secretory phenotype (SASP), fostering immunosuppressive TREM2^+^ macrophages and causing neoadjuvant immune checkpoint blockade (NICB) failure in ESCC. In preclinical models, fisetin-mediated targeting of these senescent B cells reduces cellular senescence and enhances NICB efficacy (315). Tumor-infiltrating B cells form lympho-myeloid aggregates (LMAs) that are associated with poor prognosis, whereas TLSs correlate with favorable outcomes. LMAs contain double-negative B cells (CD20^+^IgD^-^CD27^-^), which colocalize with Tregs and are associated with reduced survival (316). Collectively, these findings facilitate the rational design of therapeutic strategies that eliminate pathogenic B-cell subsets while preserving or enhancing protective humoral immune function in EC patients.

#### 4.2.2 Targeting T cells

Depleted CD8^+^ T cells constitute the predominant proliferative cell component in the TME, contributing to immunosuppression (317). Single-cell atlas of ESCC reveals dominant Tex, Tregs, and immunosuppressive myeloid cells. CD8^+^ T cells show a progression from pre-exhausted to exhausted states. Crosstalk between macrophages and Tregs contributes to immunosuppression. Targeting these T-cell-related pathways could reactivate anti-tumor immunity (57). Regarding the infiltrating CD8^+^ T cells in the TME, a T cell-mediated tumor killing (TTK)-related gene prognostic index (TTKPI) was developed for ESCC. TTKPI predicts survival and immunotherapy response, with low-risk patients benefiting more from anti-PD-L1 therapy. Key TTK gene KIF11 promotes tumor progression and inversely correlates with CD8^+^ T cell infiltration (318). Additionally, a study on NICB for ESCC identified CD8^+^ Tex-SPRY1, which are associated with improved response and prolonged survival following NICB, potentiate anti-tumor immunity, and thus represent a promising target for enhancing NICB efficacy in patients with ESCC (93). Furthermore, the combination of immunotherapy with other therapies has demonstrated promising clinical applications. Combination chemotherapy plus anti-PD-1 improves ESCC outcomes by reducing immune checkpoint interactions (TIGIT-NECTIN2, NECTIN1-CD96) between stressed tumor cells and CD8^+^ T cells, thereby preventing T-cell exhaustion. In non-responders, SLC1A3^+^ tumor cells and fibroblasts create a barrier that blocks CD8^+^ T-cell infiltration (173). Collectively, these findings underscore the necessity of multi-pronged strategies that simultaneously target CD8^+^ T cell exhaustion pathways, immunosuppressive cell networks, and tumor-intrinsic barriers to restore anti-tumor immunity in EC.

### 4.3 Targeting stromal cells in the TME of EC

#### 4.3.1 Targeting CAF cells

Studies have demonstrated that CAFs are central mediators of immunotherapy resistance in EC, so researchers have developed various therapeutic strategies to enhance the efficacy of immunotherapy by targeting CAF populations within the TME (319). Single-cell profiling has identified distinct immunosuppressive subsets that limit therapeutic efficacy. In ESCC, neoadjuvant immunochemotherapy enriches IL6^+^CCL2^+^ immunomodulatory CAFs and CD248^+^ mechanoresponsive CAFs. The former drives resistance via IL-6, whereas the latter physically impedes CD8^+^ T-cell infiltration and drug delivery (211). Besides, WNT2^+^ CAFs suppress DC differentiation and anti-tumor immunity through SOCS3/p-JAK2/p-STAT3 signaling, and WNT2 blockade restores DC function and sensitizes tumors to anti-PD-1 therapy in ESCC mouse models (210). MMP14^+^ CAFs secrete exosomal tsRNA-10522 that attenuates CD8^+^ T-cell cytotoxicity and impairs anti-PD-1 efficacy. Blocking these CAFs or their exosomal tsRNAs could restore anti-tumor immunity, positioning this subset as both a biomarker and a therapeutic target (320). Furthermore, RGS16^+^ CAFs further promote malignant progression via the NF-κB-MDK-SDC1 axis (321), while WISP1^+^ CAFs enhance ECM protein expression (COL1A1, MMP14) and promote migration and invasion of co-cultured ESCC cells (322). Given the functional heterogeneity of CAF subpopulations, future strategies should prioritize subtype-specific targeting to dismantle stromal barriers and enhance immunotherapy outcomes (323).

#### 4.3.2 Targeting TAECs

Targeting TAECs represents a novel strategy to counteract immunosuppression and potentially enhance immunotherapy efficacy (324). Single-cell profiling of EAC has revealed suppressed TAECs alongside Treg and exhausted T-cell infiltration before treatment (323). Guo et al. identified APLN^+^ TAECs driving angiogenesis in metastatic nodes, where they cooperate with MMP3^+^IL24^+^ fibroblasts and CXCL12^+^ pericytes to compose a pro-metastatic microenvironment (325). Quercetin inhibits TAEC migration and tube formation while reducing VEGFA, MMP2, and MMP9 expression, thereby exerting anti-tumor effects (326). Beyond angiogenesis, TAECs also modulate immune cell function. In ESCC, lymphatic TAECs foster accumulation of CD177^+^ Tregs, a tumor-specific subset that drives IL-35-mediated CD8^+^ T-cell exhaustion and correlates with poor survival and resistance to anti-PD-1 plus chemotherapy. Notably, this Treg population declines after treatment, suggesting that targeting TAECs may disrupt this niche and boost immunotherapy efficacy (103). However, significant phenotypic and functional heterogeneity among TAEC subpopulations, and their distinct spatial distribution patterns, remain challenges that require systematic elucidation to guide therapeutic strategies (327).

#### 4.3.3 Targeting CSCs

Targeting CSCs represents a promising therapeutic strategy in ESCC, given their pivotal role in tumor initiation, therapeutic resistance, and recurrence (328). Key stemness-regulating pathways, including Wnt/β-catenin, Notch, Hippo, and Hedgehog, drive CSC proliferation, differentiation, and self-renewal, rendering them actionable targets in ESCC (328). In preclinical models, ITGA7^high^ ESCC cells display stemness-associated gene expression and EMT features, exhibiting enhanced self-renewal, differentiation capacity, and chemoresistance. Knockdown of ITGA7 reverses these malignant phenotypes, identifying ITGA7 as both a CSC marker and a potential therapeutic target (329). Beyond eliminating existing CSC pools, therapeutic strategies should aim to block dedifferentiation or induce CSC differentiation, thereby sensitizing residual tumor cells to conventional radiotherapy and chemotherapy (330). Notably, ESCC stemness is coupled with immune evasion programs, including PD-L1 upregulation and remodeling of tumor-infiltrating immune cells. Disrupting these CSC-intrinsic regulatory circuits may therefore unlock novel immunotherapeutic and combination strategies (331). As principal drivers of tumor recurrence, metastasis, and chemoresistance, CSCs portend poor clinical outcomes. Integrating CSC-directed biomarkers with single-cell profiling offers a path to dissect intratumoral heterogeneity and design precision combination regimens that target both CSCs and their immunosuppressive niches.

### 4.4 Modulation of metabolism

Metabolic reprogramming within the TME is not merely a by-product of tumor growth but an active driver of immunotherapy resistance in EC (15). Consequently, restoring metabolic fitness to effector cells while disrupting tumor-protective adaptations represents a rational therapeutic axis (332). Targeting lactate accumulation and acidosis can relieve immune suppression. Inhibition of lactate dehydrogenase or pharmacological neutralization of extracellular acidity restores CD8^+^ T-cell and NK-cell cytotoxicity and blunts M2-like TAM polarization (15, 234). Normalizing the hypoxic niche—via HIF-1α inhibitors or anti-angiogenic agents that improve vascular perfusion—reduces PD-L1 induction and suppressive myeloid recruitment (333). Additionally, blocking immunosuppressive metabolic enzymes, including IDO1, ARG1, and the ectonucleotidases CD39/CD73, repletes arginine and tryptophan while curtailing adenosine-mediated paralysis of T and NK cells (334, 335). Finally, rewiring lipid and mitochondrial metabolism, such as inhibiting CPT1B-driven fatty-acid oxidation in PMN-MDSCs or sensitizing CSCs to ferroptosis via the Hsp27-GPX4 axis, can simultaneously weaken suppressive myeloid cells and restore lymphocyte effector function (270, 336). In ESCC, preclinical evidence supports combining these metabolic interventions with PD-1/PD-L1 blockade to convert metabolically hostile, immunosuppressive niches into permissive environments conducive to durable anti-tumor immunity.

### 4.5 Photodynamic therapy and photoimmunotherapy

Photodynamic therapy (PDT) induces immunogenic cell death, leading to the release of DAMPs and tumor-associated antigens, which promote DC maturation and subsequent CD8^+^ T cell priming (337). Phototherapy combined with immunotherapy (PIT) helps regulate immune suppression in the TME. Near-infrared phototherapy (NIR-PIT) exerts cytotoxic effects by enabling the antibody photosensitizer conjugate to specifically bind target cells following NIR laser irradiation (338). Dual-targeted NIR-PIT for EC can target epidermal growth factor receptor (EGFR) on cancer cells and fibroblast activation protein (FAP) on CAFs, thereby significantly suppressing tumor growth in mice (339). By selectively eliminating CAFs, this CAF-targeted NIR-PIT restored tumor sensitivity to 5-fluorouracil. Additionally, using an anti-FAP antibody conjugated with IR700, the therapy induced rapid and specific CAF death *in vitro* and *in vivo*, with no adverse effects (340). PDT also demonstrates potential applications in tumor immunotherapy. Talaporfin sodium photodynamic therapy (TS-PDT) is a minimally invasive salvage treatment for ESCC after chemoradiotherapy, characterized by low phototoxicity. It provides effective local control and favorable survival (341). Rescue photodynamic therapy with talaporfin sodium demonstrates efficacy in EC patients who have failed chemotherapy or radiotherapy, exhibiting a high local complete response rate (342). Additionally, a hypoxia-sensitive nanocarrier co-delivering a TOPK inhibitor and photosensitizer chlorin e6 has been developed for ESCC. The combined photodynamic-chemotherapy enables targeted laser irradiation to generate ROS, effectively stimulating NK cell infiltration and eradicating tumors while suppressing TOPK to inhibit liver metastasis and recurrence (343). Although photosensitizers such as porfimer sodium and talaporfin sodium are approved for EC, clinical PDT application remains limited by tissue penetration depth, lesion accessibility, and device integration challenges (344). Despite its high spatial precision, rigorous clinical trials are urgently needed to validate therapeutic efficacy in this setting.

### 4.6 Combination therapy and nanoparticle-mediated immunotherapy

To precisely remodel the TME while minimizing off-target toxicity, immune modulators must be selectively delivered to target cells. Nanoparticle-based combination strategies hold substantial promise for enhancing immunotherapy efficacy (345). Hyaluronic acid-modified graphene oxide nanosheets loaded with IDO1 inhibitors overcome the limited local suppression of free drugs. Combined with chimeric antigen receptor T (CAR-T) therapy, this platform blocks IDO1-mediated immunosuppression, restores T-cell function, and potentiates anti-tumor activity in ESCC (346). Aptamer EA2-modified, pH-sensitive liposomes co-encapsulating paclitaxel and luteolin enable tumor-specific uptake and stimuli-responsive release. This nanocarrier exerts synergistic anti-tumor effects, mitigates paclitaxel-induced hepatotoxicity, and remodels the immunosuppressive milieu by promoting DC maturation and T-cell infiltration in EC (347). Besides, nanomaterials exhibit intrinsic immunomodulatory capacity to reprogram TAMs (348). Ce6-SiO_2_@MnO_2_ nanoparticle-loaded stents release Mn^2+^, which shifts TAMs toward an anti-tumor phenotype via HMGB1 downregulation and STAT1 upregulation, thereby reversing immunosuppression and enhancing photodynamic therapy, ultimately improving treatment outcomes for advanced EC (349). Multiple nanotherapies are under investigation, showing strong potential in combination therapy and nanoparticle-mediated immunotherapy, as evidenced by recent approvals and mRNA vaccine advances. However, clinical translation remains hindered by toxicity, biocompatibility issues, and interspecies differences in drug diffusion (350). Establishing reliable TME-targeted delivery systems, optimizing dosing and combination strategies, and elucidating pharmacokinetic/pharmacodynamic profiles will be essential to overcome immunotherapy resistance in EC. All these therapeutic strategies for overcoming immune tolerance in EC are illustrated in **Figure 5**.

## 5 Emerging immunotherapies for EC

Beyond ICIs, several emerging modalities—including adoptive cell therapy (ACT), neoantigen vaccines, oncolytic viruses (OVs), and bispecific antibodies (BsAbs)—are being evaluated in EC (**Figure 6**) (27, 29, 351). While most remain in early-phase development (**Table 3**), they offer distinct mechanistic advantages for circumventing TME-mediated resistance.

**Figure 6:**
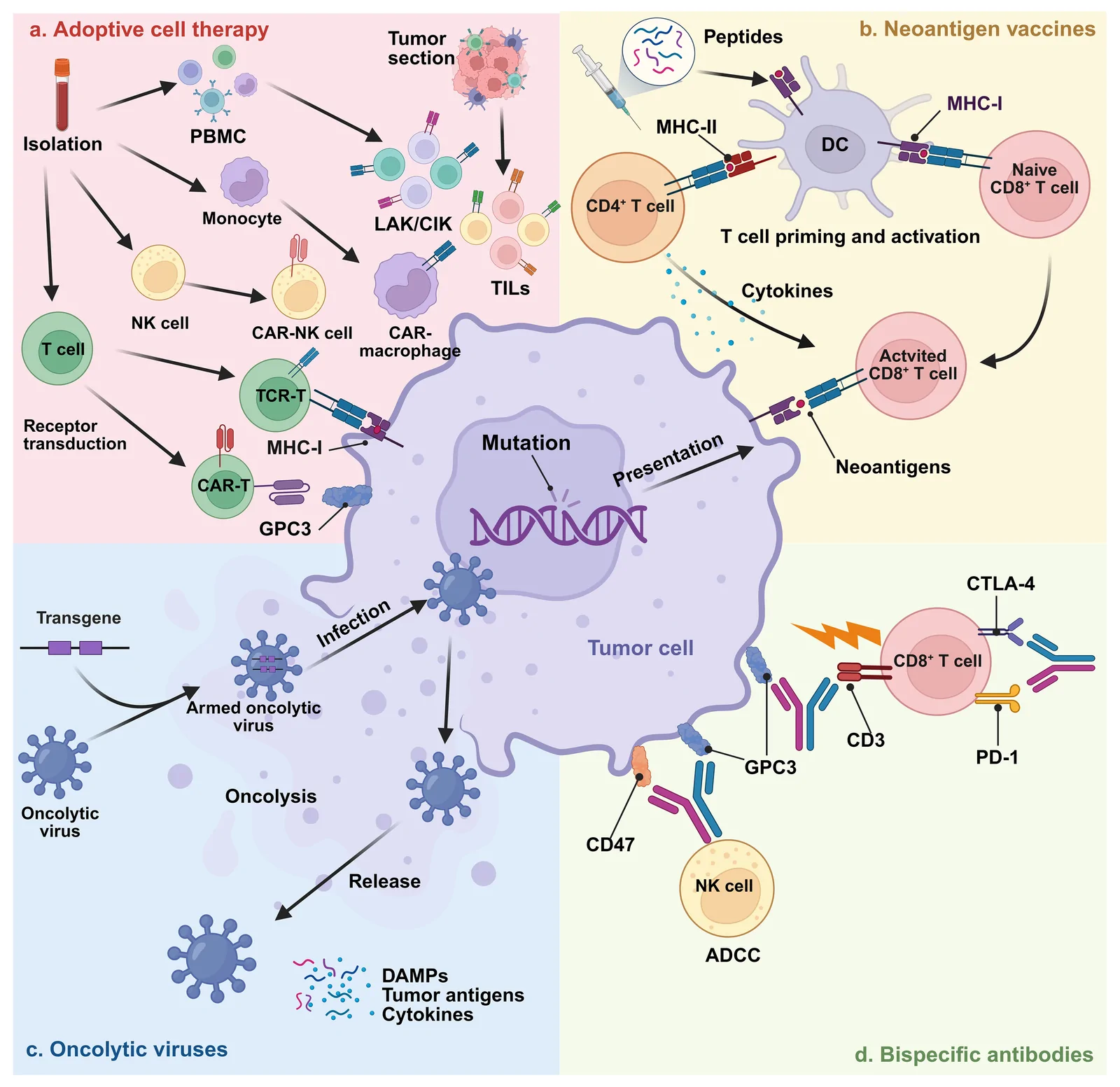
Overview of the emerging immunotherapies in EC. **(a)** Adoptive cell therapy involves the ex vivo expansion and reinfusion of immune cells, including LAK/CIK, TILs, CAR-T, and TCR-T cells, to target and eliminate tumor cells. **(b)** Neoantigen vaccines can be taken by DC cells and presented to CD4^+^ or CD8^+^ T cells through MHC molecules, leading to their activation and exerting anti-tumor effects. **(c)** Oncolytic viruses can directly lyse tumor cells or indirectly exert anti-tumor effects by inducing systemic immune responses. **(d)** Bispecific antibodies can bridge tumor cells and T cells, enabling selective destruction of tumor cells and they can also simultaneously target immune checkpoints on T cells to enhance immune responses. EC, esophageal carcinoma; PBMC, peripheral blood mononuclear cell; NK, natural killer; CAR, chimeric antigen receptor; LAK, lymphokine-activated killer; CIK, cytokine-induced killer; TIL, tumor infiltrating lymphocyte; TCR, T cell receptor; MHC, major histocompatibility complex molecule; GPC3, glypican-3; DAMP, damage-associated molecular pattern; CTLA-4, cytotoxic T-lymphocyte-associated protein 4; CD3, cluster of differentiation 3; ADCC, antibody-dependent cellular cytotoxicity.

**Table 3:** Emerging immunotherapies for esophageal carcinoma.

Therapies		ClinicalTrials.gov ID	Agents/interventions	Phase	Status
Adoptive cell therapy	TIL therapy	NCT06532799	TIL therapy combined with pembrolizumab	I/II	Recruiting
		NCT04426669	CISH gene disrupted TILs plus cyclophosphamide and fludarabine	I/II	Completed
		NCT04114136	Anti-PD-1 mAb plus metabolic modulator	II	Recruiting
		NCT02757391	CD8^+^ T cell therapy and pembrolizumab	I	Terminated
		NCT05400122	NK cells in combination with IL-2 and vactosertib	I	Suspended
	CIK cell therapy	NCT02644863	Autologous DC-CIK plus chemotherapy	II	Unknown
		NCT02490735	CIK cell therapy	II	Not yet recruiting
		NCT01691625	Chemoradiation plus DC-CIK immunotherapy	Not applicable	Completed
		NCT01691664	Radiation therapy plus DC-CIK cell therapy	Not applicable	Unknown
	CAR-T cell therapy	NCT06623396	Mesothelin-targeted CAR-T cell	I	Recruiting
		NCT05795595	Allogeneic CD70-directed CAR-T cell immunotherapy	I/II	Completed
		NCT07179692	CEA-targeted CAR-T cells	I	Recruiting
		NCT06126406		I	Recruiting
		NCT06043466		I	Recruiting
		NCT06010862		I	Recruiting
		NCT06006390		I/II	Recruiting
		NCT05539430	Claudin 18.2-targeted CAR-T cell therapy	I	Active, not recruiting
		NCT04581473		I/II	Active, not recruiting
		NCT04404595		I/II	Active, not recruiting
		NCT03874897		I	Completed
		NCT03740256	Binary oncolytic adenovirus in combination with HER2-specific autologous CAR VST	I	Recruiting
	CAR-NK cell therapy	NCT04847466	Irradiated PD-L1 CAR-NK cells plus pembrolizumab plus N-803	II	Completed
		NCT07410676	EBNK-001 allogeneic NK cells plus IL-15 plus pembrolizumab	I/II	Recruiting
	CAR-macrophage therapy	NCT04660929	Anti-HER2 CAR macrophages plus pembrolizumab	I	Unknown
	TCR T cell therapy	NCT01795976	NY-ESO-1 targeted T cells in combination with cyclophosphamide plus fludarabine and IL-2	II	Terminated
		NCT02457650	Anti-NY ESO-1 TCR-transduced T cells	I	Unknown
		NCT03941626	CAR-T/TCR-T cells immunotherapy	I/II	Unknown
		NCT02869217	NY-ESO-1 specific TCR gene transduced autologous T lymphocytes in combination with cyclophosphamide and fludarabine	I	Active, not recruiting
		NCT03159585	TCR affinity enhancing NY-ESO-1-specific T cell therapy	I	Completed
		NCT03638206	CAR-T cell immunotherapy	I/II	Unknown
		NCT03132922	Autologous genetically modified MAGE-A4^c1032^ T cells	I	Active, not recruiting
		NCT02111850	Anti-MAGE-A3-DP4 TCR peripheral blood lymphocytes	I/II	Completed
		NCT06748872	TCR-T cells plus lymphodepletion	I	Not yet recruiting
		NCT03709706	NY-ESO-1 specific TCR-T cells plus pembrolizumab	I/II	Terminated
		NCT03680560	ACTR T cell product in combination with trastuzumab	I	Terminated
Neoantigen vaccines		NCT05023928	Tumor antigen-sensitized DC vaccine	I	Completed
		NCT02693236	Adenovirus-transfected autologous DC vaccine plus CIK cells	I/II	Unknown
		NCT05317325	OCDC vaccine and NeoDC vaccine	I	Unknown
		NCT05192460	PGV002 mRNA vaccine	Not applicable	Unknown
		NCT05307835	iNeo-Vac-P01 plus GM-CSF	I	Active, not recruiting
		NCT03908671	Personalized mRNA tumor vaccine	Not applicable	Unknown
		NCT04908111	ChAdOx1 and MVA vaccines against MAGE-A3 and NY-ESO-1	I/II	Suspended
		NCT05964361	IL15-transpresenting WT1-targeted DC vaccine	I/II	Completed
		NCT03468244	Personalized mRNA tumor vaccine	Not applicable	Unknown
		NCT04041310	GAd-209-FSP and MVA-209-FSP	I/II	Active, not recruiting
		NCT01522820	DEC-205/NY-ESO-1 fusion protein CDX-1401	I	Completed
		NCT04316689	S-488210 and S-488211	I	Completed
		NCT05269381	Neoantigen peptide vaccine	I/II	Recruiting
		NCT04111172	Adenovirus 5/F35-human guanylyl cyclase C-PADRE	II	Active, not recruiting
		NCT06675201	Neoantigen-loaded DC vaccine	II	Recruiting
Oncolytic viruses		NCT06910657	IDOV-Immune (oncolytic vaccinia virus)	I	Recruiting
		NCT05830240	Recombinant oncolytic HSV1	I	Unknown
		NCT03740256	Binary oncolytic adenovirus in combination with HER2-Specific autologous CAR VST	I	Recruiting
		NCT03213054	OBP-301 in combination with radiation therapy	I	Unknown
		NCT04391049	OBP-301 given together with carboplatin, paclitaxel, and radiation therapy	I	Active, not recruiting
		NCT07061704	Oncolytic virus	I/II	Recruiting
		NCT07001592	Vaccinia virus double-deleted human IL-2	I	Recruiting
Bispecific antibodies		NCT04785820	Lomvastomig and tobemstomig compared with nivolumab	II	Completed
		NCT07481058	KC1036 in combination with PD-1 antibody and platinum	II	Recruiting
		NCT07432295	Givastomig combined with nivolumab and chemotherapy	II	Recruiting
		NCT07392892	PF-08634404 in combination with chemotherapy	II/III	Recruiting
		NCT07218003	RNDO-564 single agent or in combination with pembrolizumab	I	Enrolling by invitation
		NCT07049185	QL1706 (a dual PD-1 and CTLA-4 antibody) combined with celecoxib	II	Not yet recruiting
		NCT06746961	QL1706 plus lenvatinib	I/II	Not yet recruiting
		NCT06005493	AZD5863 monotherapy	I/II	Recruiting
		NCT05788484	CDX-585	I	Completed
		NCT04868877	MCLA-129	I/II	Active, not recruiting

### 5.1 ACT

ACT encompasses lymphokine-activated killer (LAK), cytokine-induced killer (CIK), TIL, CAR-T, and T-cell receptor-engineered T (TCR-T) cells, which are expanded ex vivo and reinfused to mediate tumor elimination (352). Early approaches such as LAK and CIK cells showed limited efficacy in solid tumors and are not further discussed here. Antigen-targeted cellular therapies, particularly CAR-T and TCR-T cells, have emerged as more promising strategies for EC.

CLDN18.2, a tight-junction protein expressed in about 25% of EAC yet largely absent from extragastric normal tissues, represents an exceptionally promising CAR-T target (353). In a phase I trial of autologous CLDN18.2-specific CAR-T cells (CT041, satri-cel) in advanced gastrointestinal cancers (NCT03874897), the ORR was 38.8% and the disease control rate was 91.8% (289). More recently, the randomized phase II CT041-ST-01 trial (NCT04581473) demonstrated that satri-cel significantly prolonged PFS versus standard therapy in previously treated CLDN18.2^+^ gastric or gastroesophageal junction cancer, marking the first randomized controlled trial of CAR-T in solid tumors (354). In ESCC, NKG2D ligands are broadly expressed and further upregulated by radiotherapy, enabling NKG2D-CAR-T-mediated tumor lysis; local radiotherapy preconditioning may enhance CAR-T infiltration by remodeling the TME (355). Nevertheless, CAR-T application in EC remains constrained by antigen heterogeneity, the immunosuppressive TME, and potential on-target toxicities (356, 357). Multiple engineering strategies are being explored to overcome these barriers in EC. CAR-T cells secreting soluble PD-1 antibody have been investigated to counteract TME-mediated exhaustion (358), and bispecific CAR-T cells co-targeting EGFR and HER3 have been developed to address antigen heterogeneity (28). Furthermore, the therapeutic potential of HER2-, EGFR-, and NKG2D-directed CAR-T cells has also been explored in preclinical and early-phase studies of esophageal and gastrointestinal malignancies (355, 359).

Unlike CAR-T cells, TCR-T cells recognize both membrane-bound and intracellular epitopes presented via MHC, enabling broader antigen targeting (360). Cancer-testis antigens are particularly suitable TCR-T targets (361). NY-ESO-1, originally identified in ESCC, is frequently co-expressed with MAGE-A4 (362, 363). In a first-in-human trial of MAGE-A4 TCR-transduced T cells for recurrent EC, engineered cells persisted for more than 5 months in half of patients, and three patients with minimal baseline tumor burden achieved long-term survival exceeding 27 months (364). High-throughput sequencing now enables the identification of neoantigen-specific T cell identification, opening avenues for personalized TCR-T therapy, although this approach remains investigational (365, 366).

### 5.2 Neoantigen vaccines

Traditional cancer vaccines based on tumor-associated antigens have shown limited clinical efficacy owing to weak immunogenicity and poor specificity (367). Neoantigens—epitopes derived from tumor-specific mutations—are recognized as “foreigners” by T cells and circumvent central tolerance as well as avoid off-target toxicity to normal tissues (368). EC exhibits a moderate-to-high TMB (median 3.5-7.5 mutations/Mb), and approximately 50% of tumors belong to a hypermutated subtype with abundant neoantigens (369, 370). Early-phase clinical trials of neoantigen vaccines in ESCC have shown only modest efficacy despite acceptable safety, highlighting the substantial barrier posed by the suppressive TME (367). Combining neoantigen vaccines with ICIs represents a rational strategy. In anti-PD-1-treated patients, both TMB and mutation-associated neoantigens positively correlate with therapeutic response (278). The randomized phase II CHANT-241 trial (NCT06675201) is evaluating maintenance neoantigen DC vaccine (Neo-DCVac) plus camrelizumab versus camrelizumab alone in unresectable locally advanced ESCC after definitive chemoradiotherapy; preliminary data indicate acceptable safety and promising anti-tumor activity (371). Direct loading of neoantigen peptides onto DCs creates DC vaccines, although inducing rapid, abundant anti-tumor T-cell responses remains challenging within the suppressive ESCC microenvironment (372, 373). Integrating genomics, transcriptomics, and immunopeptidomics may streamline neoantigen discovery and patient selection (374, 375).

### 5.3 OVs therapy

OVs selectively replicate within tumor cells to induce lysis while sparing normal tissue, concurrently triggering systemic anti-tumor immunity through the release of DAMPs and tumor antigens (376, 377). In esophageal malignancies, OBP-301 (Telomelysin), a telomerase-dependent oncolytic adenovirus, achieved a local complete response rate exceeding the pre-defined threshold in a phase II trial for advanced EC (NCT03213054), supporting its development as a locoregional therapy (378). Additionally, OH2, a recombinant herpes simplex virus 2 (HSV-2) with ICP34.5 and ICP47 deletions and armed with GM-CSF, demonstrated favorable safety and durable anti-tumor activity in metastatic EC within a multicenter phase I/II trial (NCT03866525) (379). Although OV therapy for EC remains in early clinical development, these advances underscore its substantial potential.

### 5.4 BsAbs

BsAbs simultaneously engage distinct epitopes to confer dual targeting specificity (380). Mechanistically, they are classified into two major categories: trans co-engagement, which bridges two distinct cell types (e.g., EpCAM-CD3 BsAbs redirecting T cells to selectively destroy cancer cells); and cis co-engagement, which targets two molecules on the same cell (e.g., PD-1/CTLA-4 BsAbs synergistically blocking dual checkpoints) (381). A distinctive application is the recruitment of immune effector cells to tumor cells (382). EpCAM-CD3 was among the first bispecific T-cell effector formats evaluated in solid tumors (383). Catumaxomab, an EpCAM-targeting BsAb, is approved for malignant ascites in Europe (384)—a complication frequently encountered in advanced EC. In ESCC, the EGFR×HER3 BsAb BL-B01D1 induced robust anti-tumor responses with manageable safety (28). In EAC, givastomig, a CLDN18.2/4-1BB BsAb, has shown promising clinical activity in combination with immunochemotherapy (385). Dual ICB represents another major strategy. In ESCC, the PD-L1/CTLA-4 BsAb KN046 has demonstrated promising efficacy with manageable toxicity (386), and cadonilimab has shown clinical activity and gained guideline recommendations (387). These agents offer potent immune activation with potentially lower toxicity than conventional monoclonal antibody combinations (388). Multiple clinical trials are currently evaluating PD-1/CTLA-4 BsAbs as monotherapy or in combination with chemotherapy for advanced EC (**Table 3**).

## 6 Conclusion and future perspectives

Immunotherapy has reshaped the therapeutic landscape for EC, yet durable responses remain confined to a minority of patients (9, 11, 389). As reviewed above, resistance emerges as a network property of the TME, wherein myeloid reprogramming, lymphocyte dysfunction, stromal remodeling, and metabolic dysregulation mutually reinforce one another to create an immunosuppressive niche (9, 19). Reframing resistance as a TME-driven process necessitates therapeutic strategies that concurrently target malignant cells and their surrounding ecosystem (390, 391).

Closing the translational gap remains a critical priority. While the immunosuppressive roles of MDSCs, TAMs, and Tregs are supported by correlative clinical data (57, 110), the therapeutic efficacy of reprogramming these populations rests largely on preclinical models (111, 116, 293). Conversely, the clinical activity of PD-1/PD-L1 blockade in both ESCC and EAC is established through randomized phase III trials (6, 8, 37, 42), yet the specific TME features that reliably distinguish responders from non-responders within these trials remain incompletely defined (9, 61). Therefore, future work should integrate mechanistic depth with rigorous clinical validation, explicitly calibrating the evidence level for proposed targets.

As detailed in Sections 3 and 4, pre-existing myeloid enrichment, defective antigen presentation, and dense ECM may prevent initial responses and promote primary resistance, whereas adaptive checkpoint upregulation, compensatory suppressive cell recruitment, and therapy-induced stromal remodeling drive acquired resistance after initial response. Consequently, effective strategies must simultaneously dismantle multiple, co-evolving TME barriers rather than targeting isolated populations or single axes. The profound spatial and temporal heterogeneity of the TME of EC further underscores the need to move beyond broad histological classifications. A mechanism-based, microenvironment-guided stratification is warranted: immune-desert tumors may require priming strategies (304, 377); immune-excluded tumors may benefit from stromal normalization (207, 210, 340); and inflamed yet immunosuppressed tumors may require combinations of ICI with myeloid-targeted or metabolic-modulating agents (293, 297, 346).

The integration of single-cell sequencing, spatial transcriptomics, and artificial intelligence is transforming precision immunotherapy for EC, providing unprecedented resolution of TME heterogeneity and its dynamic evolution (**Figure 7**) (94, 392–394). Emerging evidence suggests that when combined with TCR profiling and longitudinal sampling, these approaches may reveal dynamic shifts in immune cell states—such as the transition from progenitor exhausted to terminally exhausted T cells (93), compensatory upregulation of alternative checkpoints (57), or therapy-induced stromal remodeling (211)—that precede radiographic progression. Such insights could inform the development of adaptive treatment strategies and refine biomarker discovery beyond conventional metrics such as PD-L1 expression or TMB (395–397).

**Figure 7:**
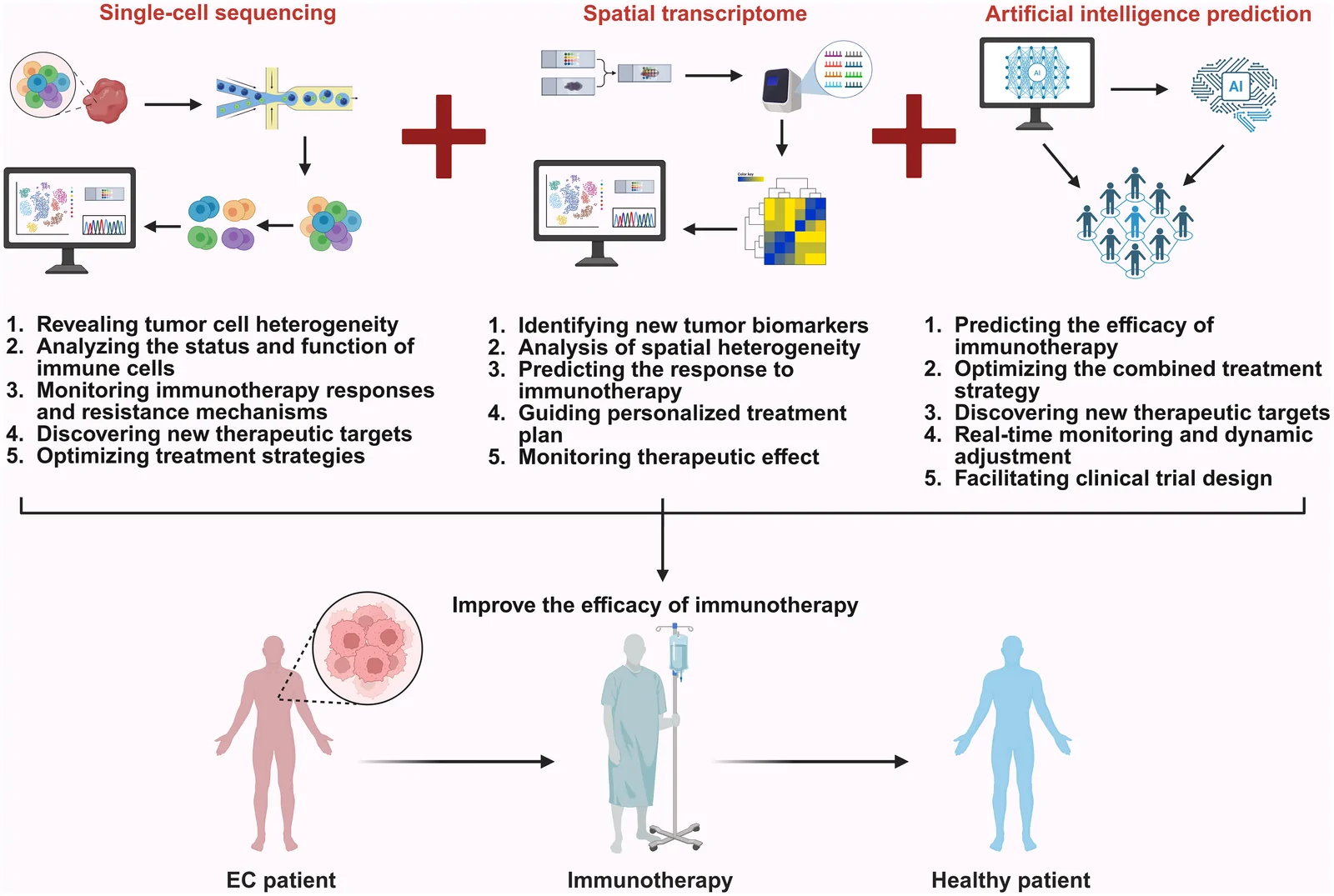
Multimodal integration of single-cell sequencing, spatial transcriptomics, and artificial intelligence transforms complex tumor-immune data into strategies for optimizing anti-tumor immunotherapy. Single-cell profiling reveals the spectrum of tumor and immune heterogeneity, distinguishing patient subsets and uncovering mechanisms that govern response or resistance. Spatial transcriptomics adds critical tissue context by mapping immune cell distribution within the tumor microenvironment and revealing whether local niches are permissive or suppressive. Artificial intelligence then synthesizes these layers to enable outcome prediction, treatment personalization, and real-time monitoring. Together, these tools allow clinicians to track treatment responses and emerging resistance, identify novel therapeutic targets, and pinpoint the rate-limiting bottlenecks—whether in immune composition, spatial architecture, or clinical predictors—that constrain immunotherapy efficacy. This convergence advances decision-making from broad prognostic grouping to precise, mechanism-guided combination therapies.

Looking forward, several priorities warrant further investigation. First, the optimal sequencing and timing of TME-directed interventions should be rigorously tested, as preclinical evidence suggests that stromal and myeloid remodeling may need to precede or accompany ICI administration to maximize efficacy (398). Second, preclinical platforms that recapitulate the stromal, vascular, and immune complexity of human EC—including patient-derived organoids, humanized xenografts, and spatially resolved ex vivo cultures—are essential to prospectively validate combination sequences (392, 396). Third, immune-related adverse events represent a critical barrier as combinations become more complex, necessitating careful toxicity monitoring and management (399, 400). Fourth, organ-specific variations in TME composition may underlie differential resistance patterns and treatment responses across anatomical sites, highlighting the need for site-specific therapeutic considerations (397, 401, 402).

In conclusion, the TME functions as an active driver of immunotherapy resistance in EC. By aligning therapeutic strategies with the specific immune phenotype of each tumor, it may become possible to convert resistant EC into an immunotherapy-responsive disease. Future efforts must prioritize multi-omic patient stratification, longitudinal TME monitoring, and the development of preclinical models to accelerate the translation of mechanism-guided combination therapies into durable clinical benefit.
